# Transcription-coupled DNA–protein crosslink repair by CSB and CRL4^CSA^-mediated degradation

**DOI:** 10.1038/s41556-024-01394-y

**Published:** 2024-04-10

**Authors:** Marjolein van Sluis, Qing Yu, Melanie van der Woude, Camila Gonzalo-Hansen, Shannon C. Dealy, Roel C. Janssens, Hedda B. Somsen, Anisha R. Ramadhin, Dick H. W. Dekkers, Hannah Lena Wienecke, Joris J. P. G. Demmers, Anja Raams, Carlota Davó-Martínez, Diana A. Llerena Schiffmacher, Marvin van Toorn, David Häckes, Karen L. Thijssen, Di Zhou, Judith G. Lammers, Alex Pines, Wim Vermeulen, Joris Pothof, Jeroen A. A. Demmers, Debbie L. C. van den Berg, Hannes Lans, Jurgen A. Marteijn

**Affiliations:** 1 Department of Molecular Genetics, Oncode Institute, Erasmus MC Cancer Institute, Erasmus University Medical Center, Rotterdam, The Netherlands; 2 Department of Cell Biology, Erasmus University Medical Center, Rotterdam, The Netherlands; 3 Proteomics Center, Erasmus University Medical Center, Rotterdam, The Netherlands

**Keywords:** Genomic instability, Nucleotide excision repair, Transcription

## Abstract

DNA–protein crosslinks (DPCs) arise from enzymatic intermediates, metabolism or chemicals like chemotherapeutics. DPCs are highly cytotoxic as they impede DNA-based processes such as replication, which is counteracted through proteolysis-mediated DPC removal by spartan (SPRTN) or the proteasome. However, whether DPCs affect transcription and how transcription-blocking DPCs are repaired remains largely unknown. Here we show that DPCs severely impede RNA polymerase II-mediated transcription and are preferentially repaired in active genes by transcription-coupled DPC (TC-DPC) repair. TC-DPC repair is initiated by recruiting the transcription-coupled nucleotide excision repair (TC-NER) factors CSB and CSA to DPC-stalled RNA polymerase II. CSA and CSB are indispensable for TC-DPC repair; however, the downstream TC-NER factors UVSSA and XPA are not, a result indicative of a non-canonical TC-NER mechanism. TC-DPC repair functions independently of SPRTN but is mediated by the ubiquitin ligase CRL4^CSA^ and the proteasome. Thus, DPCs in genes are preferentially repaired in a transcription-coupled manner to facilitate unperturbed transcription.

## Abstract (web summary)

Three studies identify a transcription-coupled DNA–protein crosslink repair pathway that depends on the Cockayne syndrome proteins and the proteasome.

## Main

DPCs are highly cytotoxic DNA lesions because their bulky nature is expected to obstruct DNA-based reactions such as replication and transcription^1,2^. Different proteins can be covalently linked to DNA through different crosslinks in either enzymatic or non-enzymatic reactions, which explains the wide structural diversity of DPCs^3^. Enzymatic DPCs consist mostly of DNA-acting proteins that form a covalent reaction intermediate with DNA during an enzymatic reaction. These are, for example, induced by chemotherapeutics such as camptothecin or etoposide that crosslink topoisomerase 1 or 2 to DNA, respectively^4,5^. 5-Aza-2′-deoxycytidine (5-Aza-dC) can also induce DPCs after incorporation into DNA during replication, which will covalently trap DNA methyltransferase 1 (DNMT1)^6^. Alternatively, non-enzymatic DPCs arise from bifunctional crosslinkers that can crosslink any protein in close proximity to DNA. These DPCs are induced by endogenously produced reactive aldehydes such as formaldehyde (FA), a by-product of, for example, histone demethylation, or acetaldehyde formed during alcohol metabolism. Although a wide variety of nuclear proteins can be crosslinked by FA, it was recently shown that mostly core histones are crosslinked^7^.

Although cells have evolved different DPC repair mechanisms, proteolytic degradation of the crosslinked protein seems to be a common feature to resolve DPCs. When a replication fork collides with a DPC, the resulting single-strand or double-strand junction is recognized by the metalloprotease SPRTN, which results in DPC removal through its protease activity^3^. Alternatively, ubiquitinylated DPCs can recruit the proteasome for replication-coupled DPC degradation, as was shown in *Xenopus* egg extracts^8^. Additionally, during replication-independent DPC repair, DPCs are SUMOylated and subsequently ubiquitinylated by the SUMO-targeted ubiquitin ligase RNF4, which results in their degradation by SPRTN or the proteasome^7,9,10^.

Thus far, most research has focused on DPC-induced replication stress owing to the resulting genome instability^3^. However, because of their bulkiness, DPCs also block transcription, as shown in in vitro transcription studies using bacteriophage T7 RNA polymerase^11,12^. Furthermore, covalently bound topoisomerase 1 complexes inhibit RNA polymerase II (Pol II)-mediated transcription and are degraded in a transcription-dependent manner by the proteasome^13,14^. These data indicate that DPCs in general might severely block transcription; however, the extent to which DPCs inhibit transcription and how such transcription-blocking DPCs are detected and subsequently repaired remains largely unknown. Recent genome-wide CRISPR–Cas9 screens have identified several genes involved in TC-NER as protective factors against FA-induced lesions^15–17^, which suggests that TC-NER might be involved in the clearance of transcription-blocking DPCs.

TC-NER removes a wide range of bulky transcription-blocking DNA lesions from the transcribed strand of active genes^18^. TC-NER is initiated when the translocase CSB (also known as ERCC6) recognizes lesion-stalled Pol II^19,20^. The TC-NER complex is then assembled through the recruitment of CSA (also known as ERCC8), which is part of the Cullin-4 RING ubiquitin-ligase complex (CRL4^CSA^), and UVSSA^21^. CRL4^CSA^ ubiquitylates CSB and elongating Pol II following DNA damage^20,22,23^. UVSSA has a dual role in TC-NER as it stabilizes CSB by recruiting the deubiquitylating enzyme USP7 (refs. ^24,25^) and promotes TFIIH recruitment^21,26^ to form the core incision complex together with XPA and RPA. After excision of the lesion by the endonucleases XPG and ERCC1–XPF, DNA polymerases refill the single-stranded DNA gap and transcription can restart^18^. Here we show that DPCs severely inhibit transcription and are preferentially repaired by a dedicated TC-DPC repair pathway, which requires CSB and CRL4^CSA^ ubiquitin ligase activity, but independent of the downstream TC-NER factors UVVSA and XPA.

## Results

### DPCs inhibit transcription

**Fig. 1:**
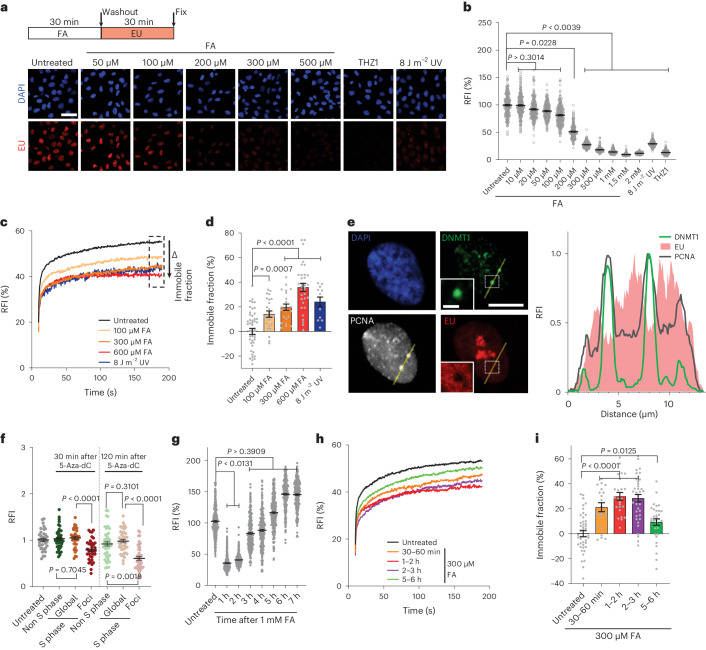
FA-induced DPCs inhibit transcription. **a**, Top: schematic of experiment. Bottom: representative images of nascent transcription levels as determined by EU pulse labelling in MRC-5 cells treated with FA, 1 μM THZ1 or UV (8 J m^–2^). Scale bar, 50 μm. **b**, Quantification of transcription levels of RNA synthesis as shown in **a**. Relative fluorescence intensities (RFI) of EU were normalized to untreated levels and set to 100%. Black lines indicate the average integrated density ± s.e.m. *n* (left to right) = 635, 754, 708, 714, 669, 594, 519, 644, 617, 545, 722, 670 and 530 cells from 3 independent experiments. Unpaired two-tailed *t*-test. **c**, FRAP analysis of GFP–Pol II using MRC-5 GFP–RPB1 KI cells untreated or 1–2 h after a 30 min FA pulse. The RFI was measured over time, background-corrected and normalized to the pre-bleach fluorescence intensity. Graphs present mean values. *n* (top to bottom) = 49, 24, 23, 33 and 23 cells from *n* = 4 (FA) or *n* = 3 (UV) independent experiments. **d**, Relative immobile fractions of GFP–Pol II calculated from data indicated in the dashed box in **c**. Values represent the mean ± s.e.m. Unpaired two-tailed *t*-test. **e**, Left: representative images of GFP–DNMT1-expressing RPE1 cells treated with a 30 min 5-Aza-dC (50 μM) pulse and fixed after 120 min. Scale bars, 10 μm and 2 μm (magnification). Right: histogram of RFI for DNMT1, PCNA and EU at the indicated line 120 min after 5-Aza-dC treatment. **f**, Quantification of EU signals at DNMT1 foci and the surrounding nucleoplasm (global) as shown in **e**. RFI values were background-corrected and normalized to untreated samples, which was set at 1. Lines show the mean ± s.e.m. *n* (left to right) = 36, 43, 36, 36, 46, 43 and 43 cells from 3 independent experiments. Unpaired two-tailed *t*-test. **g**, Quantification of recovery of transcription after FA treatment shown in Extended Data Fig. 2a. RFI values of EU were normalized to untreated levels and set to 100%. Black lines indicate the average integrated density ± s.e.m. *n* (left to right) = 626, 573, 877, 612, 601, 566, 592 and 549 cells from 3 independent experiments. Unpaired two-tailed *t*-test. **h**, GFP–Pol II FRAP as in **c** at the indicated time intervals after a 30 min FA (300 μM) pulse. Graphs represent the mean. *n* (top to bottom) = 45, 23, 24, 37 and 32 cells from 3 independent experiments. **i**, Relative immobile fractions of GFP–Pol II as in **h**. Values represent the mean ± s.e.m. Unpaired two-tailed *t*-test. Source numerical data are available in the source data. Source data

**Extended Data Fig. 1:**
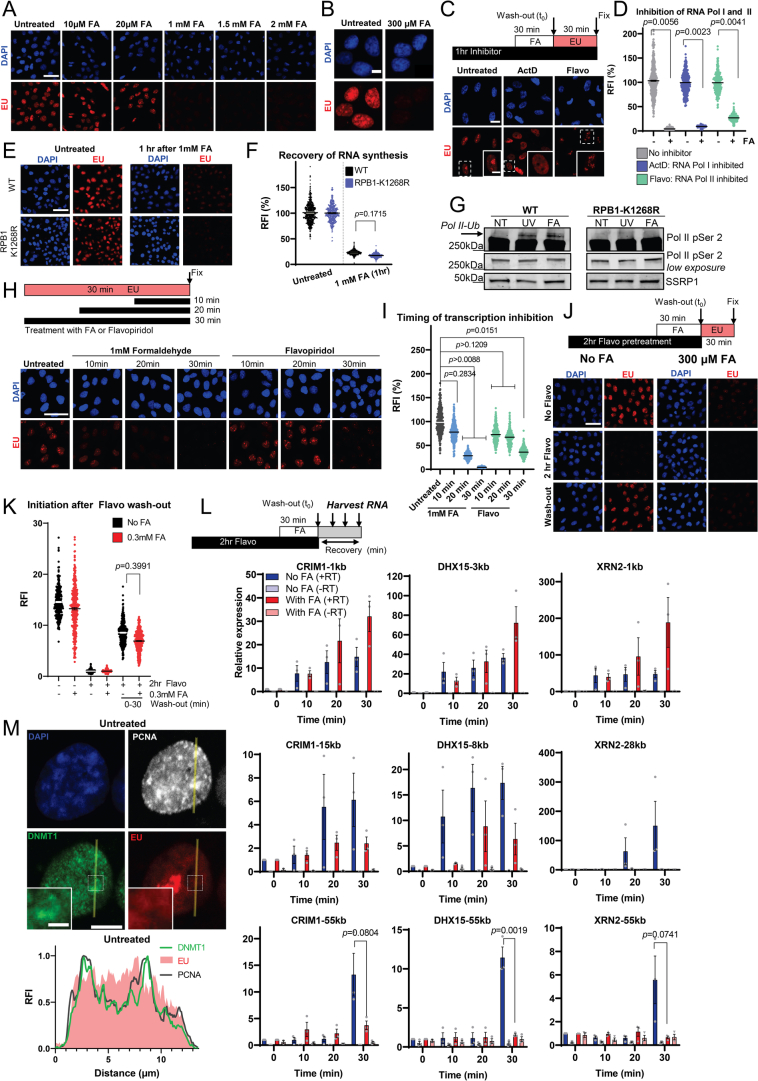
DPC-induced transcriptional inhibition requires elongating RNA Pol II. **a**: Representative images of transcription levels in MRC-5 cells treated for 30 min with the indicated concentrations of formaldehyde (FA), Quantification is shown in Fig. 1b. Scale bar = 50 μm. **b**: Close up of cells exposed to mock- or formaldehyde treatment with 300 μM FA. Scale bar = 10 μm. **c**: MRC-5 cells treated with Actinomycin D (ActD, 50 ng/ml) or Flavopiridol (Flavo, 1 μM) for 1 hr. Scale bar = 20 μm and 10 μm (magnification). **d**: MRC-5 cells, treated with the inhibitors as in (**c**), were subsequently treated with 1 mM FA for 30 min. Relative fluorescence intensities (RFI) of EU was normalized to levels without FA and set to 100%. Black lines indicate average integrated density ±S.E.M. n = 849, 1025, 745, 792, 686, 600 cells (left to right) from 3 independent experiments. Unpaired two-tailed t-test. **e**: Representative pictures of transcription levels in WT and RPB1 K1268R cells after a 1 mM FA pulse (30 min). Scale bar = 50 μm. **f**: Quantification of transcription as shown in (**b**). RFI of EU was normalized to untreated levels and set to 100%. Black lines indicate average integrated density ±S.E.M. n = 1311, 1169, 1224, 1439 cells (left to right) from 3 independent experiments. Unpaired two-tailed t-test. **g**: Immunoblot of chromatin fractions from HeLa WT and RPB1-K1268R mutant stained for pSer2 Pol II and SSRP as a loading control. This experiment was performed twice with similar results. **h**: Representative pictures of transcription levels in MRC-5 cells treated with either 1 mM FA or 1 μM Flavopiridol during a 30 min EU pulse for the indicated times. Scale bar = 50 μm. **i**: Quantification of transcription levels as shown in (**e**). RFI of EU was normalized to untreated levels and set to 100%. Black lines indicate average integrated density ±S.E.M. n = 807, 964, 852, 859, 852, 757, 829 cells (left to right) from 3 independent experiments. Unpaired two-tailed t-test. **j**: Representative pictures of transcription levels in MRC-5 cells, treated with 1 μM Flavopiridol for 2 hr prior to treatment with 1 mM FA for 30 min when indicated. Scale bar = 50 μm. **k**: Quantification of transcription levels as shown in (**j**). RFI of EU were normalized Flavopiridol-treated levels and set at 1. Black lines indicate average integrated density ±S.E.M. n = 967, 902, 937, 873, 856, 824 cells (left to right) from 3 independent experiments. Unpaired two-tailed t-test. **l**: Quantification of nascent RNA transcription after the Flavopiridol wash-out. After a 2 hr, 1 μM Flavopiridol pretreatment, MRC-5 cells were treated 1 mM FA for 30 min. After FA wash-out, RNA was collected at the indicated timepoints and the expression was assayed by RT-qPCR. Values represent the mean ±S.E.M. from 3 independent experiments. **m**: Representative images of mock-treated GFP-DNMT1 expressing RPE1 cells, which were fixed after 120 min. Right: Histogram of fluorescence signal for DNMT, PCNA and EU at the indicated line. This experiment was performed 3 times independently with similar results. Scale bar = 10 μm and 2 μm in the magnification. Source numerical data and unprocessed blots are available in source data. Source data

To investigate the transcription-inhibitory effects of DPCs, MRC-5 fibroblasts were exposed to increasing concentrations of FA and nascent transcription levels were quantified by 5-ethynyl uridine (EU) incorporation^27^. A 30 min period of FA exposure led to dose-dependent transcription inhibition. And a concentration of 300 μM FA led to a comparable level of inhibition as that of 8 J m^–2^ ultraviolet-C (UV) irradiation, a UV dose that induces a potent transcription block^28,29^ (Fig. 1a,b and Extended Data Fig. 1a). Increasing concentrations of FA led to even stronger transcription inhibition, similar to transcription inhibition with the CDK7 inhibitor THZ1 (ref. ^30^), which indicated that FA-induced DNA damage severely obstructs transcription.

Both RNA polymerase I (Pol I) transcription in nucleoli and Pol II-mediated transcription in the nucleoplasm were inhibited by FA (Extended Data Fig. 1b). Similarly, inhibition of Pol I or Pol II transcription with actinomycin D^31^ or with the CDK9 inhibitor flavopiridol^32^, respectively, followed by FA treatment (Extended Data Fig. 1c,d) confirmed that both polymerases are inhibited by DPCs. The effect of FA-induced DNA damage on Pol II-mediated transcription was also studied by measuring Pol II chromatin binding using fluorescence recovery after photobleaching (FRAP) in GFP–RPB1 knock-in (KI) cells^33^. This method enables the detection of DNA-damage-induced perturbations of elongating Pol II^34,29^. Increasing FA concentrations induced dose-dependent Pol II immobilization, similar to UV irradiation^29^, as shown by the FRAP curves (Fig. 1c) and the immobile fractions calculated from these curves (Fig. 1d).

### DPCs inhibit elongating Pol II

FA-induced transcription inhibition can be due to a direct block of elongating Pol II by DPCs or due to genome-wide transcriptional regulatory mechanisms. Several such regulatory mechanisms have been described to contribute to UV-induced transcription inhibition^18^, including Pol II degradation^35^ or inhibition of transcription initiation^35–37^. Pol II degradation did not contribute to FA-induced transcription inhibition, as similar inhibition was observed in wild-type (WT) cells and in cells expressing a RPB1(K1268R) mutant^23,35^, a lysine residue in RBP1 that is ubiquitylated following FA exposure (Extended Data Fig. 1e–g).

Next, we tested how fast transcription was inhibited following FA exposure. At 20 min after DPC induction, transcription was severely inhibited, which was quicker than inhibition by flavopiridol, in which Pol II is arrested at the promoter^38^ (Extended Data Fig. 1h,i). This result suggested that inhibition of de novo transcription initiation is not a major driver of FA-induced transcription inhibition. To exclude effects of DPCs on transcription initiation, we synchronized Pol II at the promoter with flavopiridol. Following flavopiridol washout, which restarts active transcription elongation by de novo initiation, we followed nascent transcription using EU. The results showed a modest inhibition of nascent transcription directly after FA treatment compared with undamaged conditions (Extended Data Fig. 1j,k), which indicated that de novo transcription initiation is not severely affected. This finding was confirmed by studying nascent transcription after flavopiridol washout using quantitative PCR with reverse transcription (RT–qPCR) in introns throughout the gene body of specific long genes (Extended Data Fig. 1l). Directly after Pol II promoter release by flavopiridol washout, nascent transcription was primarily detected at the beginning of genes. These transcription levels were not affected after FA exposure, which indicated that initiation is not inhibited after DPC induction. However, at later time points after flavopiridol washout, more downstream in the gene body, nascent transcription was severely inhibited, which is most likely caused by the increased probability of elongating Pol II encountering a DPC. This result indicates that transcription is mainly inhibited by the stalling of elongating Pol II at the DPC. To confirm this possibility, we treated cells expressing GFP–DNMT1 with 5-Aza-dC, thereby inducing DNMT1 crosslinks in replicated DNA, as visualized by DNMT1 foci^7,10^. By directly quantifying transcription at local DNMT1 foci, we observed a clear reduction in the EU signal, whereas EU levels outside these foci remained unaffected compared with untreated or 5-Aza-dC-treated non-S-phase cells (Fig. 1e,f and Extended Data Fig. 1m). Together, this result shows that DPC-induced transcription inhibition is mainly caused by the stalling of elongating Pol II at DPCs *in cis* rather than by transcriptional regulatory responses *in trans*.

**Extended Data Fig. 2:**
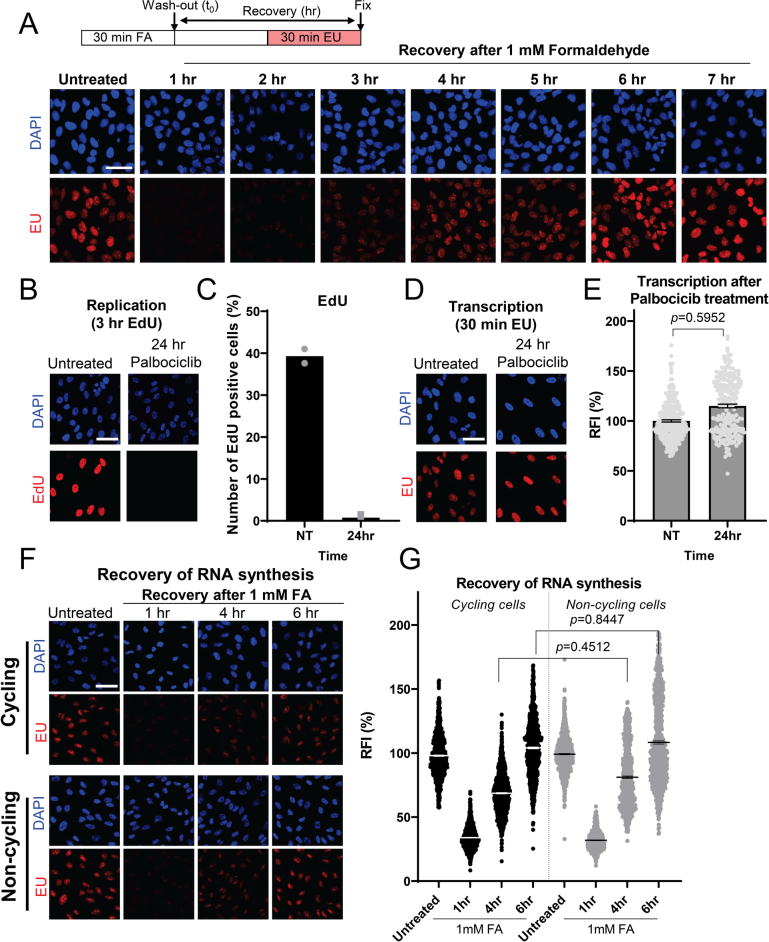
DPC-induced transcriptional inhibition is reversible independent of replication. **a**: Representative images of recovery of transcription for the indicated recovery times after a 30 min pulse of 1mM FA as determined by a 30 min pulse labelling with EU. This is quantified in Fig. 1g. Scale bar = 50 μm. **b**: Representative images of replication levels in RPE1 cells treated with 1 µM CDK4/6 inhibitor Palbociclib for 24 hr prior to pulse-labeling with EdU for 3 hr. Scale bar = 50 μm. **c**: Quantification of the number of EdU positive cells shown in (**b**). Black bars indicate average from 2 independent experiments. **d**: Representative images of transcription levels in RPE1 cells treated with 1 µM CDK4/6 inhibitor Palbociclib for 24 hr prior to EU pulse-labelling for 30 min. Scale bar = 50 μm. **e**: Quantification of the transcription levels as shown in (**d**). Relative fluorescence intensities (RFI) of EU were normalized to untreated levels and set to 100%. Black bars indicate average integrated density ±S.E.M. n = 314 or 231 cells from 2 independent experiments. Unpaired two-tailed t-test. **f**: Representative images of transcription levels in cycling and non-cycling cells. Cells were arrested with 1 µM CDK4/6 inhibitor Palbociclib for 24 hr prior to exposure to 1 mM FA for 30 min. Transcription levels were assayed by EU incorporation and visualized with click-chemistry. Scale bar = 50 μm. **g**: Quantification of the recovery of transcription in cycling cells or non-cycling RPE1 cells as shown in (**f**). RFI of EU at the indicated time points after 1 mM FA pulse treatments was normalized to untreated levels and set to 100%. Black lines indicate average integrated density ±S.E.M. n = 985, 928, 967, 974, 900, 691, 691, 752 cells (left to right) from 3 independent experiments. Unpaired two-tailed t-test. Source numerical data are available in source data. Source data

Notably, FA-induced transcriptional inhibition was rapidly reversed, as full recovery was observed within 4 h (Fig. 1g and Extended Data Fig. 2a), which indicated that these transcription-blocking lesions are quickly resolved. Similarly, whereas maximum Pol II immobilization was observed 1–2 h after FA exposure (Fig. 1h,i), Pol II chromatin binding swiftly diminished, starting 2–3 h after DPC induction. The transcription recovery after FA exposure was quicker than TC-NER-mediated transcriptional recovery after UV irradiation, which takes around 16–18 h^34^. This result suggests that FA-induced transcription blocks are resolved more efficiently or through a different repair mechanism to that of UV-induced damage. This swift repair is not caused by replication-dependent DPC repair^1,2^, as we observed similar transcription recovery in cycling cells and non-replicating cells, which were arrested in G1 using the CDK4 and CDK6 inhibitor palbociclib without inhibiting transcription^39^ (Extended Data Fig. 2b–g).

### TC-DPC repair

**Fig. 2:**
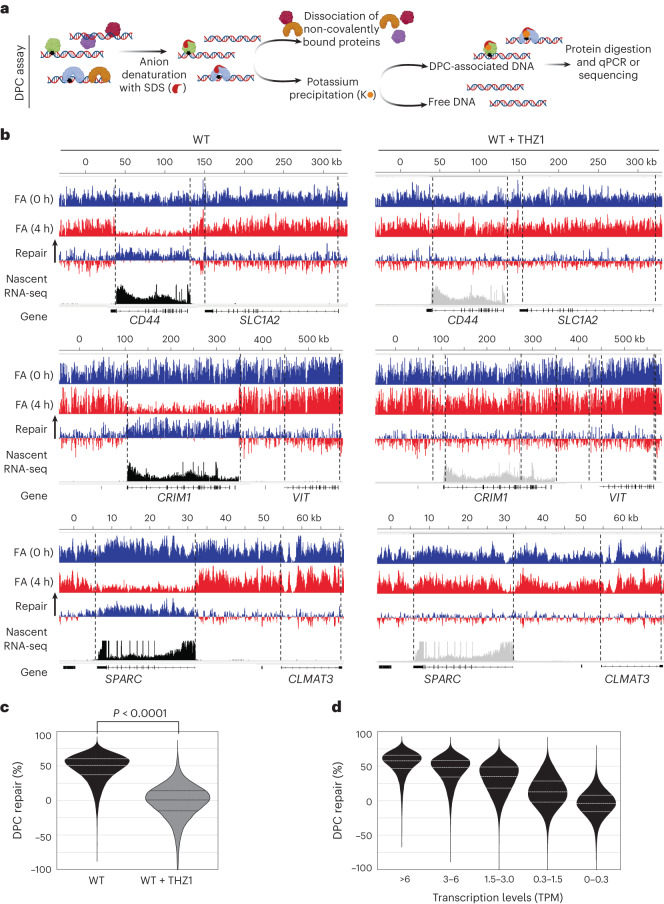
TC-DPC repair. **a**, Cartoon outlining the DPC isolation procedure. Cells were lysed in SDS, and DPCs with associated DNA were precipitated with KCl. DPC repair was determined by RT–qPCR or sequencing 0 h or 4 h after FA treatment. **b**, Left: DPC-seq reads from MRC-5 cells in representative genes directly (0 h) and 4 h after a 1 mM FA pulse of 30 min. Right: charts represent cells pre-treated with 1 μM THZ1 for 90 min before a pulse of 1 mM FA. Expressed genes were identified with nascent RNA-seq in untreated MRC-5 WT cells. Repair was calculated by subtracting 4 h from 0 h reads. **c**, For genome-wide DPC repair analysis, the genome was segmented into bins of 1 kb. DPC-seq reads were normalized per sample to non-expressed bins. Violin plots showing DPC repair in per cent (0 h – 4 h DPC-seq reads)/(0 h DPC-seq reads) in expressed bins >3 TPM. The violin plots are normalized to non-expressed bins. Median and Q1 and Q3 quartiles are plotted in the violin plots, and values represent data from two independent DPC-seq experiments. Unpaired two-tailed *t*-test. **d**, Violin plot as described in **c**, whereby DPC repair per cent in WT MRC-5 cells is binned on the indicated expression levels. Schematic in **a** was created using BioRender (https://www.biorender.com).

**Extended Data Fig. 3:**
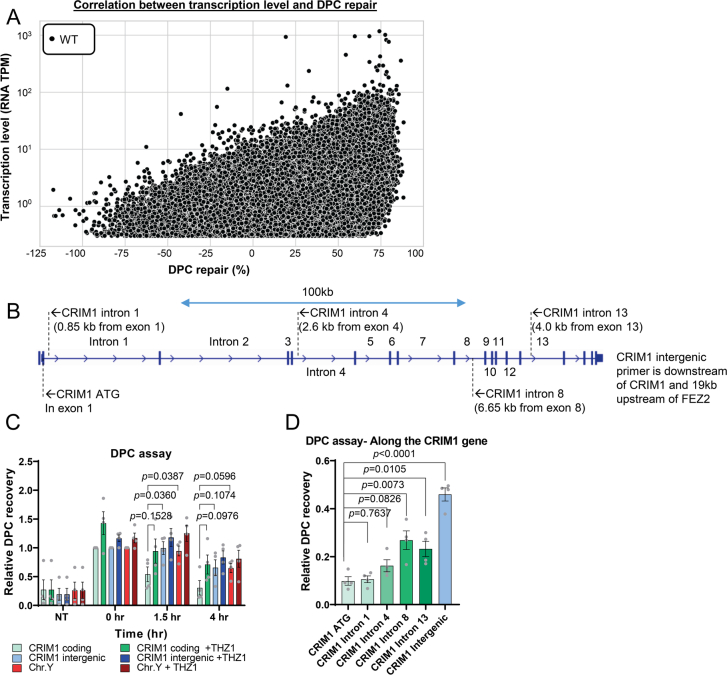
DPC repair is more efficient across actively transcribed genes. **a**: DPC repair % in expressed bins was plotted against their expression level. Spearman correlation between DPC repair and transcription levels was 0.61997. **b**: A graphic representation of the RT-qPCR primers used in the DPC removal assay along the CRIM1 gene. **c**: Relative DPC recovery in untreated (NT) or after the indicated times after a 30 min 1 mM FA pulse using K-SDS precipitation followed by qPCR of genomic DNA in the CRIM1 gene and CRIM1 intergenic region as well a silent genomic region on chromosome Y. Values were normalized to 0 hr conditions for each primer set at 1 and represent mean with S.E.M. from 4 independent experiments. **d**: Relative DPC recovery at 4 hr after a 30 min 1 mM FA pulse using K-SDS precipitation followed by qPCR of genomic DNA in the CRIM1 gene and CRIM1 intergenic region. Values were normalized to 0 hr conditions (not shown) for each primer set at 1 and represent mean with S.E.M. from 4 independent experiments. Source numerical data are available in source data. Source data

It was notable that this transcription recovery after FA treatment was faster than the repair of total cellular DPCs observed before^40^, which implied that these transcription-blocking DPCs are preferentially repaired. To test this possibility, we isolated DPCs by removing non-crosslinked proteins from the DNA by denaturation, after which DPCs were separated from free DNA by K-SDS precipitation^41,42^ (Fig. 2a). The DPC-associated DNA was analysed using next-generation sequencing. DPC reads were equally distributed in both genic and intergenic regions directly after FA induction (0 h). By contrast, after a 4 h recovery period, DPCs were strongly reduced in the representative genes *CRIM1*, *CD44* and *SPARC*, which are actively transcribed as determined by nascent RNA sequencing (RNA-seq)^43^ (Fig. 2b, left). Of note, this preferential DPC repair was not observed in the neighbouring non-expressed genes *VIT*, *SLC1A2* and *CLMAT3*, which indicated that DPC repair in genes depends on active transcription. Preferential DPC repair in actively transcribed genes depended fully on transcription, as THZ1 pre-treatment completely abolished preferential DPC repair in active genes (Fig. 2b, right). Transcription-dependent DPC repair was confirmed by qPCR on the DPC-associated DNA in the *CRIM1* gene (Extended Data Fig. 3b,c). Notably, performing this analysis at different regions from 5′ to 3′ in *CRIM1* showed that repair at the 5′ end was more efficient (Extended Data Fig. 3d).

To test whether TC-DPC repair of actively transcribed genes was a genome-wide response, we divided the DPC sequencing (DPC-seq) results in bins of 1 kb and analysed DPC repair in expressed bins (>3 transcripts per million base pairs (TPM)). This analysis showed that ~50% of all DPCs in these bins were repaired within 4 h, which could be fully inhibited by transcription inhibition (Fig. 2c). This transcription dependency for TC-DPC repair was confirmed by the fact that TC-DPC repair efficiency was directly linked to expression levels. That is, in genes expressed at low levels, TC-DPC repair was slower than in genes expressed at higher levels (Fig. 2d and Extended Data Fig. 3a). Together, these data show that a transcription-coupled repair mechanism exists to preferentially repair DPCs in active genes, and the transcription dependency indicates that elongating Pol II is involved in damage recognition.

### TC-DPC repair factors

**Fig. 3:**
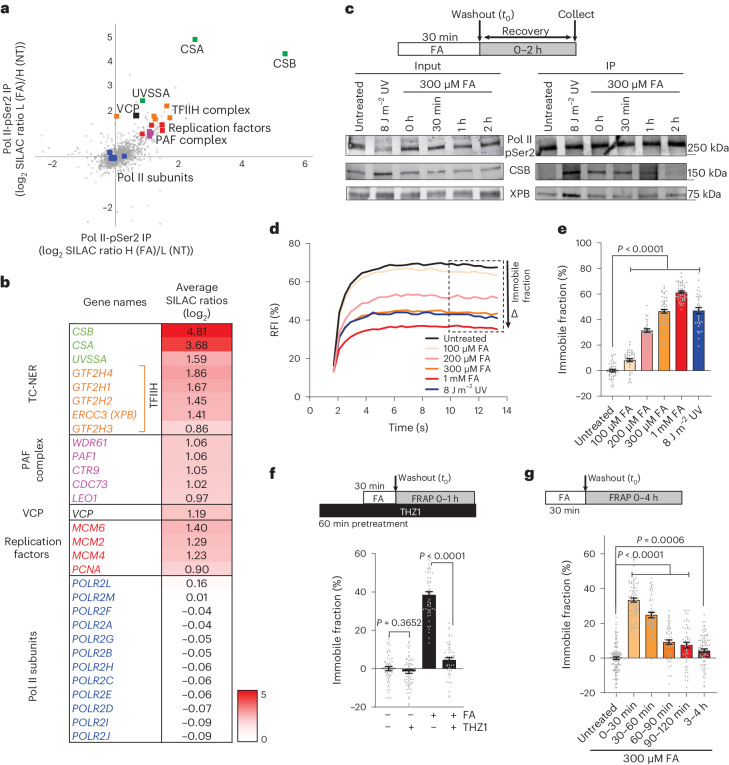
TC-NER factors are recruited to DPC-stalled Pol II. **a**, Scatter plot of log_2_ stable isotope labelling by amino acids in cell culture (SILAC) ratios of SILAC-based quantitative interaction proteomics of pSer2-modified RPB1 across two independent experiments, including a label swap. Pol II-interacting proteins were compared between mock-treated cells and cells treated with 300 μM FA for 45 min. TC-NER proteins are depicted in green and Pol II subunits in blue. IP, immunoprecipitation; NT, untreated; H, heavy; L, light. **b**, Heatmap of interacting proteins of DPC-stalled elongating Pol II based on the average SILAC ratios (log_2_) as shown in **a**. **c**, Top: scheme of experiment. Bottom: IP of pSer2-modified Pol II followed by immunoblotting for the indicated proteins over time after a 30 min pulse of 300 μM FA and collected either directly after the pulse (0 h) or allowed to recover for the indicated times. Pol II interactions were compared with cells collected 1 h after UV (8 J m^–2^) irradiation. This experiment was performed twice with similar results. **d**, CSB–mScarlet-I FRAP with indicated doses of FA compared with 8 J m^–2^ UV irradiation. Cells were imaged 30 min after FA exposure without washout. The lines represent the mean. *n* (top to bottom) = 43, 41, 30, 52, 49 and 34 cells from 3 independent experiments. **e**, Relative immobile fractions of mScarlet-I–CSB FRAP as in **c**. Values represent the mean ± s.e.m. Unpaired two-tailed *t*-test. **f**, Top: scheme of experiment. Bottom: relative immobile fractions of CSB FRAP in cells pre-treated with 1 μM THZ1 before exposure to 300 μM FA. Values represent the mean ± s.e.m. *n* (left to right) = 52, 64, 52 and 54 cells from 3 independent experiments. Unpaired two-tailed *t*-test. **g**, Top: scheme of experiment. Bottom: relative immobile fractions of mScarlet-I–CSB FRAP in cells treated with 30 min of a 300 μM FA pulse and followed in time. Values represent the mean ± s.e.m. *n* (left to right) = 122, 82, 52, 56, 53 and 87 cells from 3 independent experiments. Unpaired two-tailed *t*-test. Source numerical data (**d**–**g**) and unprocessed blots (**c**) are available in the source data. Source data

**Extended Data Fig. 4:**
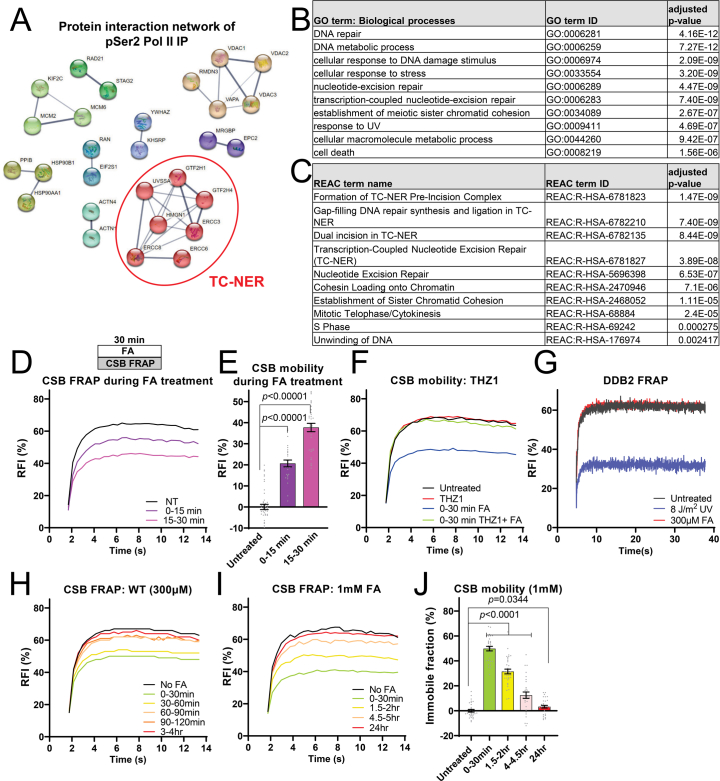
DPC-stalled Pol II is bound by TC-NER factors. **a**: STRING analysis of the top hits shown in Fig. 3b. The cluster of TC-NER is highlighted by a red cycle. **b**: Top 10 enriched GO terms (biological process) identified using g:Profiler of FA- enriched proteins that bind to elongating Pol II genes. **c**: Top 10 enriched GO terms (reactions) identified using g:Profiler of FA- enriched proteins that bind to elongating Pol II genes. **d**: CSB-mScarlet-I FRAP during the 30 min FA pulse of 300 μM. The graph represents an average ±S.E.M. n = 45, 27, 31 cells (up to down) from 3 independent experiments. **e**: Relative immobile fraction is shown in (**d**). Values represent mean ±S.E.M. Unpaired two-tailed t-test. **f**: CSB-mScarlet-I FRAP pretreated with 1 μM THZ1 and subsequently with 300 μM of FA for 30 min. The graph represents an average ±S.E.M. n = 52, 64, 52, 54 cells (up to down) from 3 independent experiments. Relative immobile fraction is shown in Fig. 3f. **g**: GFP-DDB2 FRAP with 300 μM of FA for 30 min or 8 J m^-2^ UV-C irradiation. The graph represents an average ± S.E.M. n = 64, 64, 67 cells (up to down) from 3 independent experiments. **h**: CSB-mScarlet-I FRAP with 300 μM of FA for 30 min. The graph represents an average ±S.E.M. n = 122, 82, 52, 56, 53, 87 cells (up to down) from 3 independent experiments. Relative immobile fraction is shown in Fig. 3g. **i**: CSB-mScarlet-I FRAP with 1 mM of FA for 30 min. The graph represents an average ±S.E.M. n = 32, 31, 35, 30, 33 cells (up to down) from 2 independent experiments. **j**: Relative immobile fractions of mScarlet-I-CSB FRAP as in (**I**). Values represent mean ±S.E.M. Unpaired two-tailed t-test. Source numerical data are available in source data. Source data

To determine which proteins and repair pathways are involved in TC-DPC repair, we performed quantitative interaction proteomics and compared the interactors of phosphoSer2 (pSer2)-modified elongating Pol II in unperturbed conditions with those after FA-induced damage (Fig. 3a and Supplementary Table 1). Gene ontology (GO) analysis revealed an enrichment of proteins particularly involved in TC-NER (Extended Data Fig. 4a–c). The top DPC-induced Pol II interactors were the TC-NER initiation factors CSA and CSB. More downstream TC-NER factors such as UVSSA and TFIIH were also enriched, but to a lesser extent (Fig. 3b and Extended Data Fig. 4a). Furthermore, the Pol II interaction with the PAF complex was increased, which has been described to have a role in transcription recovery after UV-induced damage^44^. Replication factors were also enriched, a result indicative of possible DPC-induced transcription-replication conflicts^45^.

CSB recognizes lesion-stalled Pol II during TC-NER^19,20^. Our proteomics data indicated that this also happens after DPC induction, which was confirmed by immunoprecipitation of elongating Pol II that showed a clear FA-induced CSB interaction (Fig. 3c). In contrast to CSB, only a minor FA-induced Pol II interaction with the TFIIH subunit XPB was detected, which was much weaker than upon UV-induced damage. This result suggests that FA-induced damage might be repaired in a mechanistically distinct manner to that of UV-induced damage.

The CSB interaction with DPC-stalled Pol II was further studied by FRAP using CSB–mScarlet-I KI cells, which is a sensitive live-cell imaging method to measure the interaction of CSB with lesion-stalled Pol II^46^. CSB–mScarlet-I FRAP showed strong and dose-dependent immobilization after FA treatment, with 300 µM of FA resulting in a similar CSB immobilization level as 8 J m^–2^ UV irradiation (Fig. 3d,e). This FA-induced CSB immobilization was swiftly detected following FA exposure (Extended Data Fig. 4d,e) and happened in a transcription-dependent manner (Fig. 3f and Extended Data Fig. 4f). This result excludes the possibility that CSB immobilization is caused by direct FA-induced crosslinking of CSB to chromatin. The global-genome nucleotide excision repair (GG-NER) damage sensor DDB2 was not immobilized after FA treatment, which confirmed the specificity of the response of CSB to FA (Extended Data Fig. 4g). In line with the timing of the loss of Pol II–CSB interaction (Fig. 3c), CSB immobilization was almost completely recovered 2 h after FA exposure, a result indicative of repair of DPCs (Fig. 3g and Extended Data Fig. 4h). Of note, the timing of the recovery of CSB mobility was dose-dependent, as in cells treated with 1 mM FA, CSB mobility recovered after ~4 h (Extended Data Fig. 4i,j), which is similar to the timing of transcription recovery after 1 mM FA treatment (Fig. 1g). Collectively, these results show that the TC-NER factors CSB and CSA in particular are recruited to DPC-stalled Pol II, which suggests that these TC-NER factors are involved in TC-DPC repair.

### TC-DPC repair relies on non-canonical TC-NER

**Fig. 4:**
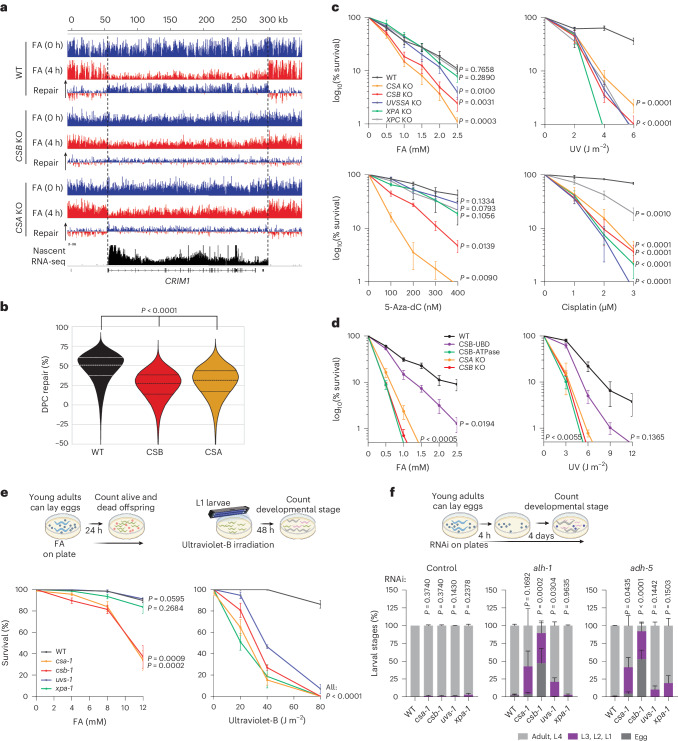
Transcription-coupled repair of DPCs by CSA and CSB. **a**, DPC-seq reads from MRC-5 WT, *CSA* and *CSB* KO cells in the *CRIM1* gene directly (0 h) and 4 h after a 30 min pulse of 1 mM FA as described in Fig. 2b. **b**, Violin plots of per cent of DPC repair in expressed bins (>3 TPM) in MRC-5 WT, *CSA* and *CSB* KO cells as described in Fig. 2c. Unpaired two-tailed *t*-test. **c**, Relative colony survival of MRC-5 WT and indicated TC-NER KO cells treated with the indicated doses of FA (1 h pulse), UV, 5-Aza-dC (continuous) or cisplatin (1 day pulse). Graphs represent the mean ± s.e.m. from *n* = 7 (FA) or *n* = 3 (UV, 5-Aza-dC and cisplatin) independent experiments. Graphs were normalized to the untreated colony number, which was set at 100%. Unpaired two-tailed *t*-test with values from 2.5 mM FA, 6 J m^–2^ UV, 400 nM 5-Aza-dC and 3 μM cisplatin. **d**, Relative colony survival assay of HCT116 WT, CSB mutants and TC-NER KO cell lines treated with 1 h of FA pulse (left) or UV (right). The endogenous KI of CSB(K538R) disrupts ATPase activity, whereas the CSB-GG mutant (L1427G and L1428G) has a deficient UBD. Graphs were normalized to the untreated colony number, which was set at 100%. Graphs represent the mean ± s.e.m. *n* = 3 independent experiments. Unpaired two-tailed *t*-test using values from 1 mM FA and 3 J m^–2^ UV. **e**, Top: scheme of procedure. Bottom: FA (left) and ultraviolet-B irradiation (right) survival assays of WT, *csa-1*, *csb-1*, *uvs-1* and *xpa-1* mutant *C.* *elegans*. Shown is the mean ± s.e.m. of three independent experiments. Unpaired two-tailed *t*-test using values from 12 mM FA and 80 J m^–2^ UV. **f**, Top: scheme of procedure. Bottom: developmental growth of WT, *csa-1*, *csb-1*, *uvs-1* and *xpa-1* mutant *C.* *elegans* after control RNA interference (RNAi) or depletion of *alh-1* or *adh-5*. For each condition, the developmental stage was counted as ‘adult, L4’, ‘L3, L2, L1’ or ‘egg’. Shown is the average ± s.e.m. of three independent experiments. Unpaired two-tailed *t*-test using values from ‘adult, L4’. Source numerical data are available in the source data. Schemes in **e** and **f** were created using BioRender (https://biorender.com). Source data

**Extended Data Fig. 5:**
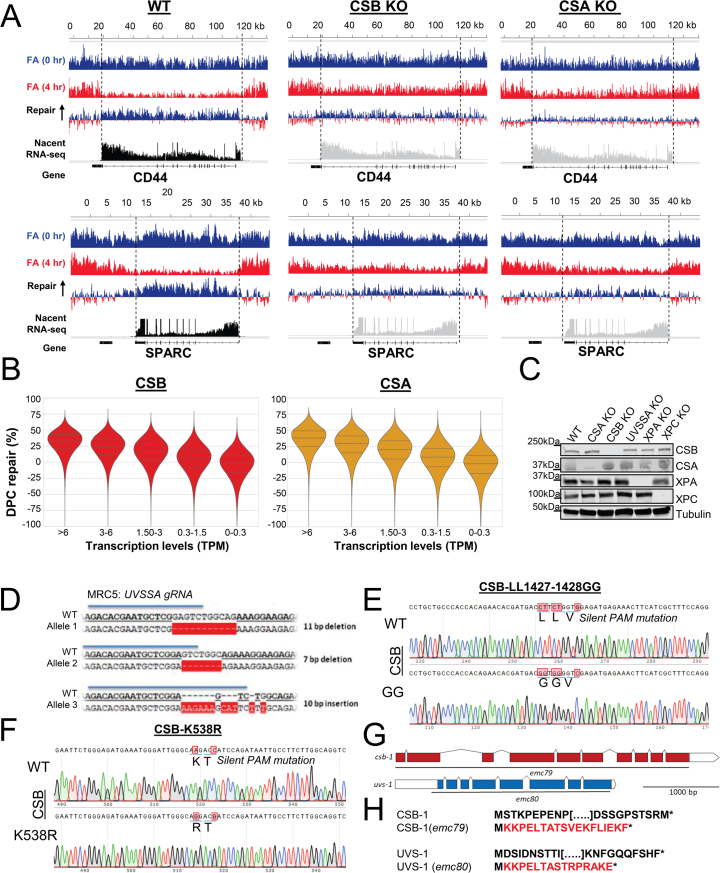
CSA and CSB are required for transcription-coupled DPC repair. **a**: Representative images of DPC-Seq at 0 hr (blue) and 4 hr (red) after a 1 mM FA pulse of 30 min in MRC-5 WT, CSA and CSB KO cells. Repair is calculated by subtracting 4 hr from 0 hr. Expressed genes were identified by nascent RNA-Seq. **b**: Violin plots showing DPC repair in MRC-5 WT, CSA and CSB KO cells in expressed genes, sorted into indicated bin based on their expression levels. In the violin plots are normalized to non-expressed genes. In the plots, the median and Q1 and Q3 quartiles are plotted. **c**: Immunoblot for the indicated proteins in various MRC-5 GFP-RPB1 KI cells with KO of NER factors. This experiment was performed twice with similar results. **d**: Schematic representation of MRC-5 GFP-RPB1 KI UVSSA KO cells. **e**: Sequencing of HCT116 CSB-LL1427-28GG knock-in clone, which disrupts the ubiquitin binding domain (UBD). **f**: Sequencing of HCT116 CSB-K538R knock-in clone, which disrupts the ATPase activity of CSB. **g**: Schematic depiction of the *csb-1* locus (top) and *uvs-1* locus (bottom) under which the knockout alleles *emc79* and *emc80* showing full removal of both genes are indicated. **h**: Predicted peptide sequences encoded by alleles *emc79* (top) and *emc80* (bottom) are shown as compared to the N- and C-terminal protein sequences of the wild type CSB-1 and UVS-1 proteins. Red color indicated nonsense sequence. Unprocessed blots are available in source data. Source data

To test the functional relevance of the TC-NER factors CSB and CSA in the repair of DPCs, we performed DPC-seq in *CSB* and *CSA* knockout (KO) cells (Fig. 4a and Extended Data Fig. 5a,b). TC-DPC repair was markedly reduced in *CSB* and *CSA* KO cells compared with WT cells, as shown in representative genes and by a genome-wide analysis of transcribed genes (Fig. 4a,b and Extended Data Fig. 5a,b). This result indicates that CSB and CSA have crucial roles in TC-DPC repair.

Subsequently, we performed colony survival experiments of TC-NER KO cell lines in response to a 1 h pulse of FA. Whereas all TC-NER KO cells showed an equal sensitivity to UV irradiation, only *CSA* and *CSB* KO cells displayed a strong hypersensitivity to FA (Fig. 4c and Extended Data Fig. 5c,d). Notably, *UVSSA* KO cells were only mildly sensitive to FA, especially at higher concentrations, whereas *XPA* KO cells showed a similar sensitivity as WT cells and cells deficient for the GG-NER factor XPC. These data suggest that mainly CSA and CSB are involved in TC-DPC repair, whereas UVSSA has only a minor contribution and XPA is not involved, which indicates that not all TC-NER factors are equally important for resolving DPC-stalled Pol II. Similar results were obtained following the induction of enzymatically induced DPCs. *CSB* and *CSA* KO cells were hypersensitive to 5-Aza-dC, which crosslinks DNMT1 to DNA without inducing interstrand and intrastrand DNA crosslinks^7,10,47^, whereas *UVSSA* and *XPA* KO cells showed similar survival rates as WT cells (Fig. 4c). Mutations in the ATPase-binding domain and ubiquitin-binding domain (UBD) of CSB showed that both domains were equally important after UV-induced and FA-induced damage (Fig. 4d and Extended Data Fig. 5e,f), which suggests that CSB performs a similar function during TC-NER and TC-DPC repair. TC-NER KO cells were equally sensitive to cisplatin, which mainly creates interstrand and intrastrand crosslinks^48^ (Fig. 4c), a result that excludes the possibility that the CSA and CSB-dependent effects observed after FA exposure are due to these types of DNA–DNA crosslinks. Together, these data show that although all TC-NER factors are crucial for the repair of UV-induced and cisplatin-induced transcription-blocking DNA damage, a non-canonical TC-NER mechanism that specifically involves CSB and CSA is important for TC-DPC repair.

These results were confirmed in the multicellular model organism *Caenorhabditis elegans*, in which TC-NER is highly conserved^49,50^. TC-NER-deficient mutant animals showed similar UV hypersensitivity. However, similar to survival in human cells, FA hypersensitivity was only observed for *csa-1* and *csb-1* worms, but not for *uvs-1* and *xpa-1* animals (Fig. 4e and Extended Data Fig. 5g,h). We also used *C.* *elegans* to study the effects of DPCs induced by endogenous aldehydes produced by cellular metabolism. We depleted the *alh-1* and *adh-5* homologues of the human aldehyde dehydrogenase X (ALDH1B1) and alcohol dehydrogenase 5 (ALDH5) enzymes, respectively, which are required for detoxification of aldehydes^51–53^. Accumulation of endogenous aldehydes had no effect on the survival of WT animals, whereas *csa-1 and csb-1* animals showed strong developmental arrest following depletion of either *alh-1* or *adh-5* (Fig. 4f)*. uvs-1* and *xpa-1* animals developed only mild phenotypes after depletion of the aldehyde dehydrogenases, which could be attributed to DNA crosslink sensitivity^54^.

### CSB and CSA are crucial for transcription restart

**Fig. 5:**
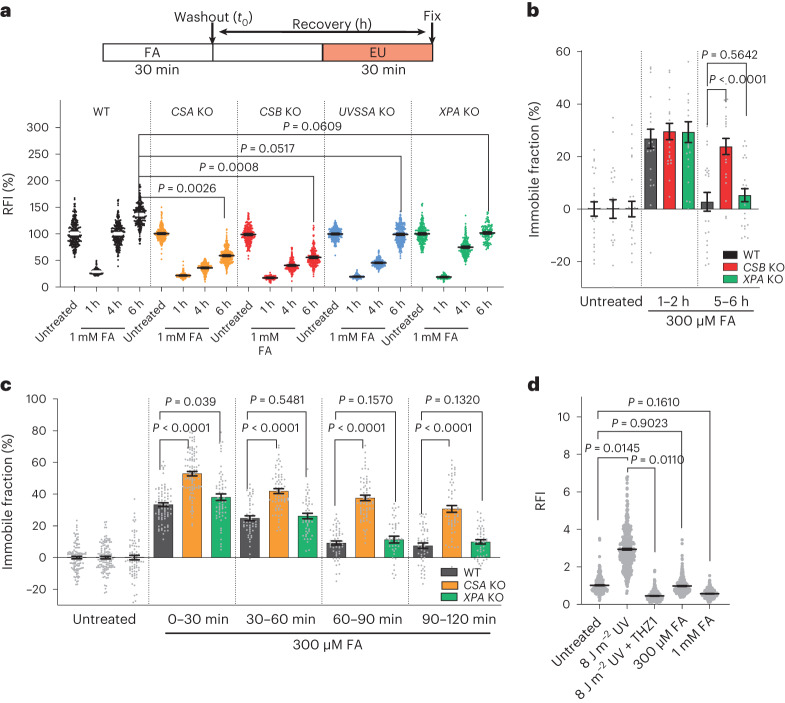
DPCs are repaired by non-canonical TC-NER. **a**, Top: scheme of experiment. Bottom: quantification of recovery of transcription after a 30 min pulse of 1 mM FA. RFI of EU were normalized to untreated levels and set to 100%. Black lines indicate the average integrated density ± s.e.m. *n* (left to right) = 779, 683, 759, 758, 801, 647, 676, 786, 692, 524, 697, 615, 696, 625, 609, 673, 368, 277, 364 and 334 cells from 3 independent experiments. Unpaired two-tailed *t*-test at the 6 h time point. **b**, Relative immobile fractions of GFP–Pol II in MRC-5 GFP–RPB1 KI WT, *XPA* KO or *CSB* KO cells at the indicated time intervals after a 30 min pulse with 300 μM FA. Values represent the mean ± s.e.m. *n* (left to right) = 23, 23, 26, 24, 22, 22, 25, 22 and 24 cells from 3 independent experiments. Unpaired two-tailed *t*-test. **c**, Relative immobile fractions of CSB FRAP after a 30 min pulse with 300 μM FA followed over time in KO cells. Values represent the mean ± s.e.m. *n* (left to right) = 122, 129, 82, 82, 96, 61, 52, 67, 47, 56, 68, 44, 53, 57 and 48 cells from 3 (WT and *XPA* KO) or 4 (*CSA* KO) independent experiments. Unpaired two-tailed *t*-test. **d**, TCR-UDS in non-replicating RPE1 *XPC* KO by 1 μM palbociclib treatment for 24 h before treatment with the indicated doses of FA or with 8 J m^–2^ UV. Cells were pre-treated with 2 μM THZ1 where indicated. Values represent the mean ± s.e.m. *n* (left to right) = 648, 997, 502, 754 and 610 cells from 3 independent experiments. Unpaired two-tailed *t*-test. Source numerical data are available in the source data. Source data

**Extended Data Fig. 6:**
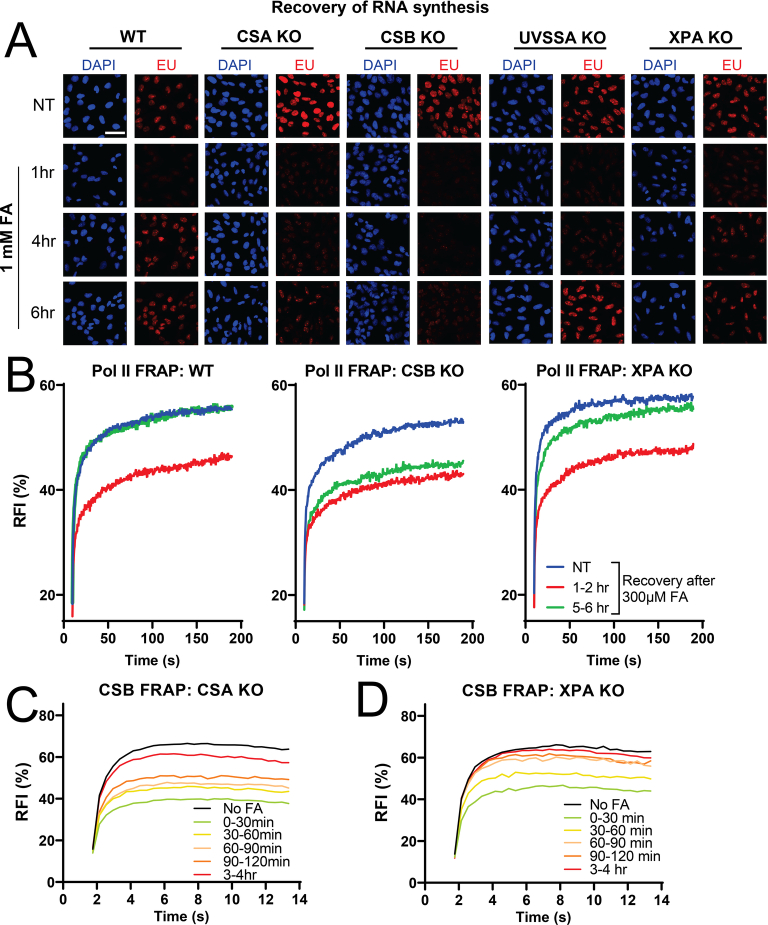
TC-DPC repair requires TC-NER factors CSB, but not downstream XPA. **a**: Representative images of transcription levels in MRC-5 KO cells treated for 30 min with 1 mM FA as determined by EU pulse-labelling for 30 min. Quantification is shown in Fig. 5a. Scale bar = 50 μm. **b**: GFP-Pol II FRAP in MRC-5 GFP-RPB1 KI WT of XPA and CSB KO cells at the indicated time-intervals after a pulse treatment with 300 μM FA for 30 min. Line represent mean of n = 23, 24, 25 cells (WT), n = 23, 22, 22 cells (CSB), n = 26, 22, 24 cells (XPA) (up to down) from 3 independent experiments. Relative immobile fraction as shown in Fig. 5b. **c**: CSB-mScarlet-I FRAP with in HCT116 CSA KO cells treated with 300 μM FA for 30 min. Graph is an average of n = 129, 96, 67, 68, 57, 87 cells (up to down) from 4 independent experiments. Relative immobile fraction as shown in Fig. 5c. **d**: CSB-mScarlet-I FRAP with in HCT116 XPA KO cells treated with 300 μM FA for 30 min. Graph is an average of n = 82, 61, 47, 44, 48, 98 cells (up to down) from 4 independent experiments. Relative immobile fraction as shown in Fig. 5c. Source numerical data are available in source data. Source data

As CSA and CSB are important for TC-DPC repair, we established their role in the recovery of transcription following DPC induction. Transcription was fully recovered in WT cells 4 h after FA exposure, whereas this recovery was almost completely abolished in *CSA* and *CSB* KO cells (Fig. 5a and Extended Data Fig. 6a). Transcription in *XPA* KO cells recovered to a similar degree as in WT cells, whereas *UVSSA* KO cells showed a slight delay in transcription recovery, but fully recovered after 6 h. To confirm the CSB-dependent transcription recovery effect, we assessed Pol II and CSB chromatin binding by FRAP. In WT and *XPA* KO cells, Pol II mobility recovered to undamaged conditions within 5–6 h after FA, whereas in *CSB* KO cells, Pol II remained immobilized to a similar extent as directly after FA exposure (Fig. 5b and Extended Data Fig. 6b). Similarly, in *XPA* KO cells, CSB mobility recovered with similar kinetics as in WT cells, whereas in *CSA* KO cells, we observed a prolonged immobilization of CSB, which persisted for up to 2 h (Fig. 5c and Extended Data Fig. 6c,d). Together, these results indicate that only CSA and CSB are essential to resolve DPC-stalled Pol II, whereas for canonical TC-NER, UVSSA and XPA are also crucial.

In TC-NER, UVSSA recruits TFIIH^21,26^, which together with XPA is crucial for DNA damage verification and subsequent DNA damage excision. However UVSSA and XPA are not essential for TC-DPC repair, which indicated that DPCs are not removed by NER-mediated excision followed by DNA synthesis to fill the single-stranded DNA gap^18^. To test this hypothesis, we quantified transcription-coupled repair-mediated unscheduled DNA synthesis (TCR-UDS) using 5-ethynyl-2′-deoxyuridine (EdU) incorporation in non-replicating GG-NER-deficient cells^55^. Although a clear transcription-dependent TCR-UDS signal was observed after UV exposure, no TCR-UDS signal was detected after FA treatment, not even at higher concentrations (Fig. 5d). This result indicates that during TC-DPC repair, no major DNA synthesis steps occur and that TC-DPC repair is therefore mechanistically distinct from canonical TC-NER.

**Extended Data Fig. 7:**
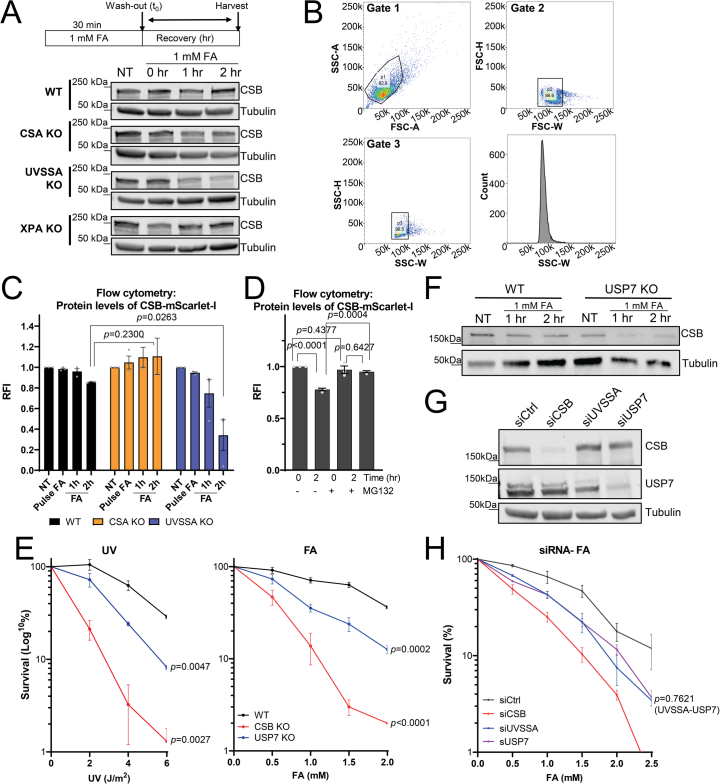
UVSSA and USP7 are required to prevent CSB degradation. **a**: CSB immunoblot of indicated HCT116 KO cells treated with 1 mM FA for 30 min and harvested at the indicated times. Tubulin was used as a loading control. This experiment was performed twice with similar results. **b**: Representative image of flow cytometry gating of CSB-mScarlet to determine CSB protein levels. Gating was used to exclude dead cells based on SCC-A/FSC-A plots and single cells were gated based on FSC-H/FSC-W and subsequently SSC-H/SSC-W. SSC- Side Scatter Channel, FSC- Forward Scatter Channel, A-Area, H-Height, W-Wight. **c**: Flow cytometry of CSB-mScarlet after a pulse of 1 mM FA for 30 min in the indicated cell lines. Cells were harvested at the indicated times and the fluorescent levels were determined by flow cytometry. Values were normalized to untreated levels and represent the average ±S.E.M. from 3 independent experiments. Unpaired two-tailed t-test. **d**: Flow cytometry of CSB-mScarlet after a pulse of 1 mM FA for 30 min in the presence of proteasome inhibitor MG132. Cells were harvested at the indicated times and the fluorescent levels were determined by flow cytometry. Values were normalized to untreated levels and represent the average ±S.E.M. from 3 independent experiments. Unpaired two-tailed t-test. **e**: Relative colony survival of U2OS KO cells treated with the indicated doses of FA for 1 hr and UV. Graph represents the mean with ±S.E.M. from 3 independent experiments. Unpaired two-tailed t-test at 6 J m^-2^ UV and 2 mM FA. **f**: Immunoblotting of cells after FA, stained for CSB and Tubulin. U2OS WT and USP7 KO cells were treated with 1 mM FA prior to harvesting at the indicated times. This experiment was performed twice with similar results. **g**: Immunoblot of siRNA transfected lysates of HeLa cells, stained with antibodies against CSB, USP7 and Tubulin. This experiment was performed twice with similar results. **h**: Relative colony survival of siRNA transfected HeLa cells treated with the indicated doses of FA for 1 hr. Graph represents the mean with ±S.E.M. from 3 independent experiments. Unpaired two-tailed t-test at 2.5 mM FA between UVSSA- and USP7-depleted cells. Source numerical data and unprocessed blots are available in source data. Source data

Notably, although UVSSA is not essential for TC-DPC repair, it enhanced DPC repair, as shown by delayed transcription recovery (Fig. 5a) and intermediate FA sensitivity in the absence of UVSSA (Fig. 4c). In addition to recruiting TFIIH^26^, in TC-NER, UVSSA stabilizes CSB through USP7 (refs. ^24,26^). As no repair synthesis was observed during TC-DPC repair, UVSSA is probably not needed to recruit TFIIH, but is probably only needed to stabilize CSB through USP7. Indeed, in *UVSSA* KO cells, we observed a clear FA-induced proteasomal degradation of CSB, which was not observed in WT, *CSA* or *XPA* KO cells (Extended Data Fig. 7a–d). Furthermore, similar to *UVSSA* KO cells, *USP7* KO cells^56^ showed increased CSB degradation and intermediate hypersensitivity to FA-induced DPCs, levels that were similar to those observed in *UVSSA* KO cells (Extended Data Fig. 7e–h). This result suggests that UVSSA is not essential but stimulates TC-DPC repair by stabilizing CSB through USP7 recruitment.

### TC-DPC repair is independent of SPRTN

**Fig. 6:**
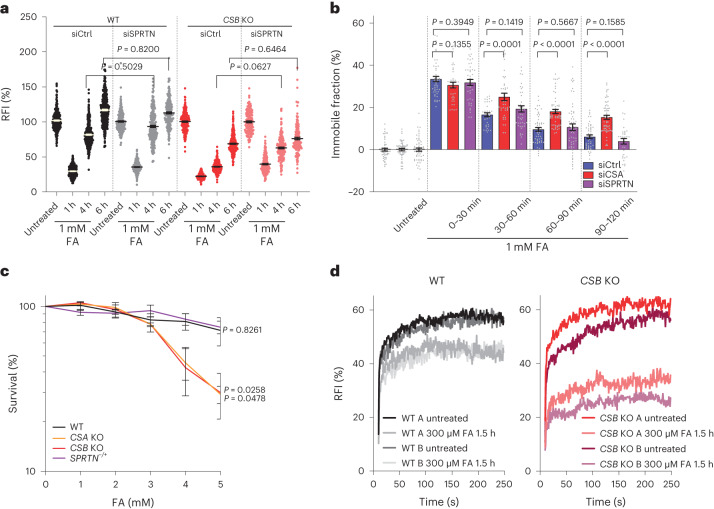
SPRTN-independent TC-DPC repair. **a**, Quantification of recovery of transcription in RPE1 WT and *CSB* KO cells transfected with short interfering RNA (siRNA) targeting *SPRTN* (siSPRTN) or a control siRNA (siCtrl) after a 30 min pulse of 1 mM FA. After FA washout, cells were left to recover for the indicated times, including a 30 min pulse labelling with EU before fixation. RFI of EU were normalized to untreated levels and set to 100%. Black lines indicate the average integrated density ± s.e.m. from three independent experiments. *n* (left to right) = 777, 634, 742, 757, 509, 481, 491, 458, 494, 480, 448, 616, 333, 371, 356 and 349 cells. Unpaired two-tailed *t*-test. **b**, Relative immobile fraction of CSB FRAP in non-replicating RPE1 CSB–mScarlet-I cells transfected with the indicated siRNAs and treated with 1 μM palbociclib 24 h before FRAP. Cells were treated with 300 μM FA for 30 min and followed for the indicated times. Values represent the mean ± s.e.m. from three independent experiments. *n* (left to right) = 49, 51, 55, 46, 37, 41, 34, 39, 45, 55, 56, 55, 46, 60 and 34 cells. Unpaired two-tailed *t*-test. **c**, Relative survival of non-replicating RPE1 cells as determined by Alamar Blue staining. RPE1 cells, arrested in G1 by treatment with 1 μM palbociclib, were treated with the indicated doses of FA for 1 h and allowed to recover for 4 days. Metabolic activity was assayed as a measure of cell viability. Values represent the mean ± s.e.m. from four independent experiments and were normalized to untreated cells. Unpaired two-tailed *t*-test at 5 mM FA. **d**, GFP–Pol II FRAP in WT and *CSB* KO neurons. GFP–RPB1 KI iPS cells were differentiated into post-mitotic neurons through neurogenin-2 induction. FRAP in two different clones (A and B) was performed after treatment with 300 μM FA for 90 min. Graph represents values from eight cells. Source numerical data are available in the source data. Source data

**Extended Data Fig. 8:**
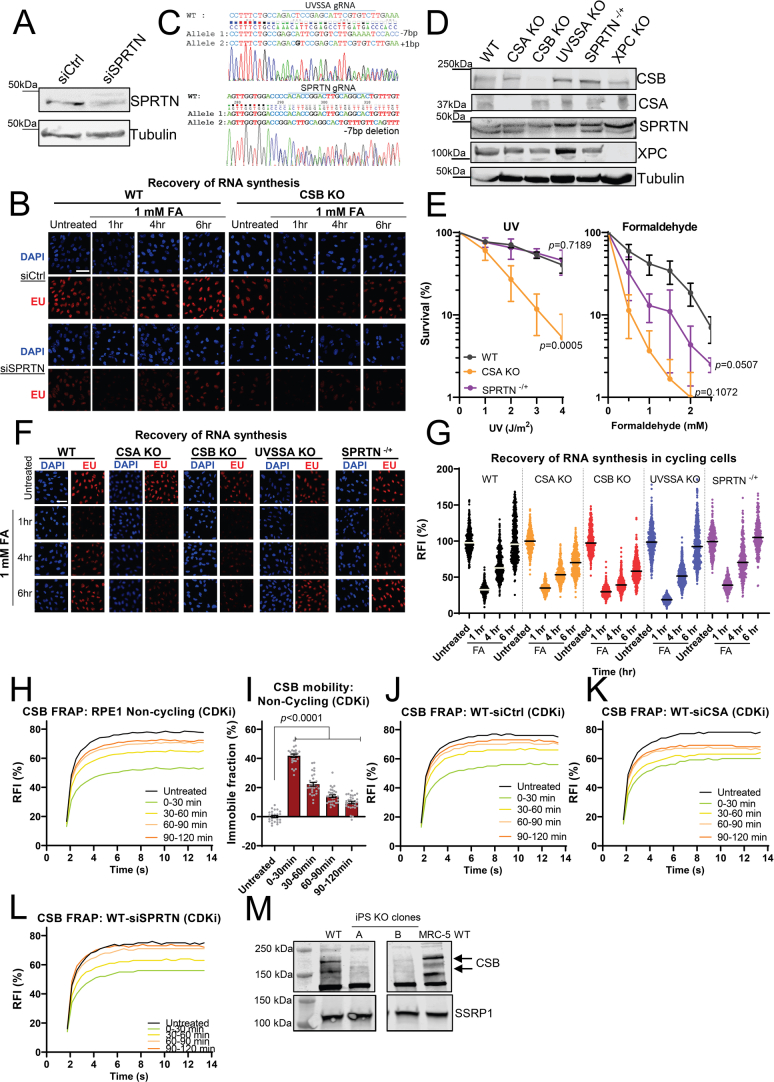
TC-DPC repair is independent of SPRTN. **a**: Immunoblot of RPE1 cells, transfected with siRNA against SPRTN, stained with antibodies against SPRTN and Tubulin. This experiment was performed twice with similar results. **b**: Representative pictures of transcription levels in RPE1 WT and CSB KO cells transfected with siRNAs against SPRTN. Quantification is shown in Fig. 6a. Scale bar, 50 = μm. **c**: Sequencing of UVSSA and SPRTN mutations in RPE1 cells. **d**: Immunoblot for the indicated proteins in various RPE1 KO cell lines. This experiment was performed twice with similar results. **e**: Clonogenic survival assay in RPE1 cells after varying doses of UV and FA. Graph represents the average ±S.E.M. from 3 independent experiments. Unpaired two-tailed t-test at 3 J m^-2^ UV and 2 mM FA. **f**: Representative pictures of transcription levels in WT, CSA KO, CSB KO, UVSSA KO and SPRTN ^−/+^ RPE1 cells. Scale bar = 50 μm. **g**: Quantification of the transcription levels as shown in (**f**). Relative fluorescence intensities (RFI) of EU were normalized to mock-treated levels and set to 100%. Black lines indicate average integrated density ±S.E.M. n = 424, 407, 408, 654, 352, 308, 345, 345, 420, 385, 493, 413, 508, 482, 576, 626, 302, 283, 294, 304 cells (left to right) from 2 independent experiments. **h**: CSB-mScarlet-I FRAP with in cells non-replicating cells which were treated with 1 μM Palbociclib (CDKi) for 24 hr prior to FRAP after 1 mM FA for 30 min. Graph is an average of n = 35, 37, 40, 40, 40 cells (up to down) from 4 independent experiments. **i**: Relative immobile fractions of mScarlet-I-CSB FRAP as in (**h**). Values represent mean ± S.E.M. Unpaired two-tailed t-test. **j**: CSB-mScarlet-I FRAP with in cells transfected with control (Ctrl) siRNA. Cells were treated with 1 μM Palbociclib (CDKi) for 24 hr prior to FRAP after 1 mM FA for 30 min. Relative immobile fraction as shown in Fig. 6b. Graph is an average of n = 49, 46, 34, 55, 46 cells (up to down) from 3 independent experiments. **k**: CSB-mScarlet-I FRAP with in cells transfected with siCSA RNA. Cells were treated with 1 μM Palbociclib (CDKi) for 24 hr prior to FRAP after 1 mM FA for 30 min. Relative immobile fraction as shown in Fig. 6b. Graph is an average of n = 51, 37, 39, 56, 60 cells (up to down) from 4 independent experiments. **l**: CSB-mScarlet-I FRAP with in cells transfected with siSPRTN RNA. Cells were treated with 1 μM Palbociclib (CDKi) for 24 hr prior to FRAP after 1 mM FA for 30 min. Relative immobile fraction as shown in Fig. 6b. Graph is an average of n = 55, 41, 45, 55, 34 cells (up to down) from 3 independent experiments. **m**: Characterization of the CSB mutation in iPS GFP-RPB1 cells. This experiment was performed twice with similar results. Source numerical data and unprocessed blots are available in source data. Source data

As the TC-DPC repair pathway does not require TC-NER-mediated excision of DPCs, we tested whether the DPC protease SPRTN is involved in this pathway. Depletion of SPRTN in either WT or *CSB* KO cells did not affect transcription recovery, which indicated that SPRTN is not involved in TC-DPC repair (Fig. 6a and Extended Data Fig. 8a,b). Moreover, SPRTN-haploinsufficient RPE1 cells, which accumulate DPCs^57^ and are hypersensitive to FA in clonogenic survival assays (Extended Data Fig. 8c–e), showed no defect in transcription recovery (Extended Data Fig. 8f,g). SPRTN is involved in both replication-dependent^1,2^ and global-genome repair of FA-induced DPCs^7^, whereby the latter is considered to be mainly active in S/G2 cells owing to low SPRTN protein levels in G1 phase^7,58^. TC-DPC repair was fully active in non-cycling cells, as shown by the similar transcription recovery kinetics after FA treatment in G1-arrested and in cycling cells (Extended Data Fig. 2g). Similarly, CSB remobilization in non-cycling cells was SPRTN-independent, but was delayed after CSA depletion (Fig. 6b and Extended Data Fig. 8h–l).

This result suggests that TC-DPC repair represents the predominant DPC repair pathway in non-replicating or differentiated cells. To test the contribution of TC-DPC repair in non-replicating cells, we performed Alamar-Blue-based survival assays, which showed that non-replicating CSA and *CSB* KO cells were hypersensitive to FA, whereas SPRTN-haploinsufficient cells showed a similar sensitivity as WT cells (Fig. 6c). Additionally, we tested whether in in vitro differentiated, post-mitotic neurons, FA-induced damage results in lesion-stalled Pol II, which needs to be cleared by TC-DPC repair. GFP–RPB1 KI induced pluripotent stem (iPS) cells were differentiated into post-mitotic neurons through neurogenin-2 induction^59,60^, and Pol II mobility was determined by FRAP. Pol II was immobilized after FA treatment in WT neurons, which was substantially increased in the absence of CSB (Fig. 6d and Extended Data Fig. 8m). Together, these data indicate that in non-cycling and differentiated cells, TC-DPC repair is one of the major repair pathways to resolve DPC-stalled Pol II and thereby stimulates cell survival in a process independent of SPRTN.

### Pol II degradation after DPCs is not mediated by CSB or CSA

**Fig. 7:**
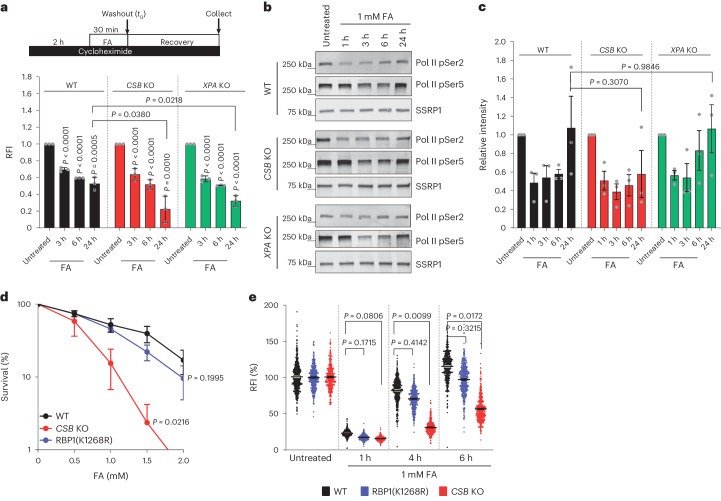
DPC repair is independent of Pol II degradation. **a**, Top: scheme of experiment. Bottom: relative GFP–RPB1 protein levels measured by flow cytometry in the presence of 100 µM cycloheximide after a pulse of 1 mM FA for 30 min. RFI of GFP were normalized to mock-treated levels and set to 1. Bars represent the mean fluorescence ± s.e.m. from three independent experiments. Unpaired two-tailed *t*-test. **b**, Chromatin-bound elongating Pol II as determined by immunoblotting with the indicated antibodies. MRC-5 cells were treated with 1 mM FA for 30 min and collected at the indicated times. SSRP1 was used as the loading control. This experiment was performed three times with similar results. **c**, Quantification of pSer2-modified RPB1 levels as shown in **b**. Values indicate the average integrated density ± s.e.m. from three independent experiments. Unpaired two-tailed *t*-test. **d**, Relative survival of WT, *CSB* KO or K1268R mutated RPB1 HeLa cells treated with the indicated doses of FA for 1 h. Values represent the mean ± s.e.m. from three independent experiments. Unpaired two-tailed *t*-test at 1.5 mM FA. **e**, Quantification of recovery of transcription in WT, *CSB* KO or K1268R mutated RPB1 HeLa cells after a 30 min pulse of 1 mM FA. After FA washout, cells were left to recover for the indicated times, including a 30 min pulse labelling with EU. RFI of EU were normalized to mock-treated levels and set to 100%. Black lines indicate the average integrated density ± s.e.m. *n* (left to right) = 1,311, 1,169, 1,162, 1,224, 1,439, 1,360, 1,236, 1,386, 973, 1,286, 1,310 and 1,107 cells from 3 independent experiments. Source numerical data (**a**,**c**–**e**) and unprocessed blots (**b**) are available in the source data. Source data

**Extended Data Fig. 9:**
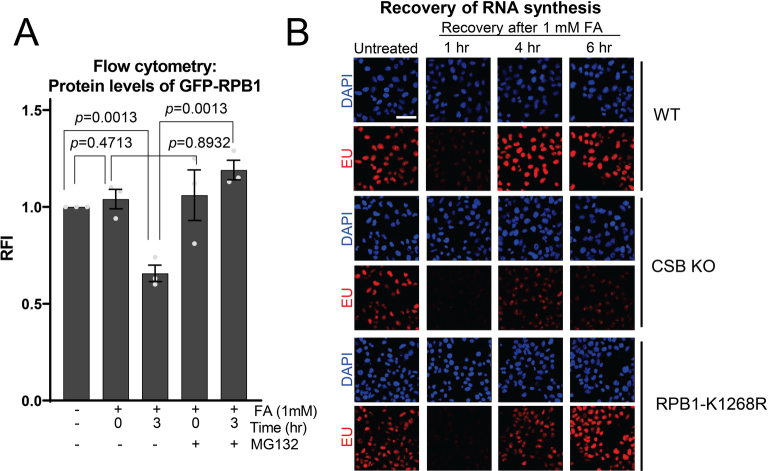
Pol II degradation is not required for DPC repair. **a**: Flow cytometry of GFP-RPB1 after a pulse of 1 mM FA for 30 min in the presence of proteasome inhibitor MG132. Cells were harvested at the indicated times and the fluorescent levels were determined by flow cytometry. Values were normalized to untreated levels and represent the average ±S.E.M. from 3 independent experiments. Unpaired two-tailed t-test. **b**: Representative pictures of transcription levels in WT, CSB KO and RPB1 K1268R cells, which are quantified in Fig. 7e. Scale bar = 50 μm. Source numerical data are available in source data. Source data

In TC-NER, CSB binds to lesion-stalled Pol II and recruits CSA, which is part of the ubiquitin ligase CRL4^CSA^ that has been shown to ubiquitylate elongating Pol II following transcription-blocking DNA damage^18^. After DPC induction, Pol II remains chromatin-bound for a prolonged time in *CSB* KO cells (Figs. 5b and 6d), which suggests that degradation of lesion-stalled Pol II by CRL4^CSA^ is required to resolve DPC-stalled elongation Pol II. To test this hypothesis, we studied Pol II half-life in WT cells by quantifying GFP–RPB1 fluorescence levels by flow cytometry at different time points after FA exposure and in the presence of cycloheximide and compared this to its half-life in *CSB* and *XPA* KO cells. Following FA treatment, a similar level of proteasomal degradation of Pol II was observed in *CSB* KO cells and in WT and *XPA* KO cells (Fig. 7a and Extended Data Fig. 9a). This FA-induced loss of Pol II probably represents the degradation of DPC-stalled elongating Pol II. To test this possibility, we performed cell fractionation assays and quantified pSer2-modified elongating Pol II in the chromatin fraction. pSer2-modified Pol II was degraded in WT, *CSB* and *XPA* KO cells (Fig. 7b,c). Notably, in contrast to *CSB* KO cells, elongating Pol II levels recovered at later time points in WT and *XPA* KO cells, which can be explained by the activity of TC-DPC repair, which subsequently results in transcription recovery and Pol II resynthesis.

During TC-NER, elongating Pol II is mainly ubiquitylated on K1268 in RPB1, which is crucial for TFIIH recruitment and subsequent repair and results in Pol II degradation^23,35^. However, both clonogenic survival and transcription recovery in RPB1(K1268R) cells were not severely affected after FA exposure (Fig. 7d,e and Extended Data Fig. 9b), which indicated that Pol II ubiquitylation is not crucial for the response to FA. Together, our data show that Pol II is degraded after FA; however, this degradation is not dependent on CSB or CRL4^CSA^. This finding suggests that the prolonged chromatin binding of elongating Pol II observed in *CSB* KO cells is not due to the lack of Pol II degradation mediated by CRL4^CSA^, but is most likely to be caused by the absence of TC-DPC repair.

### CRL4^CSA^ complex-mediated clearance of DPCs

**Fig. 8:**
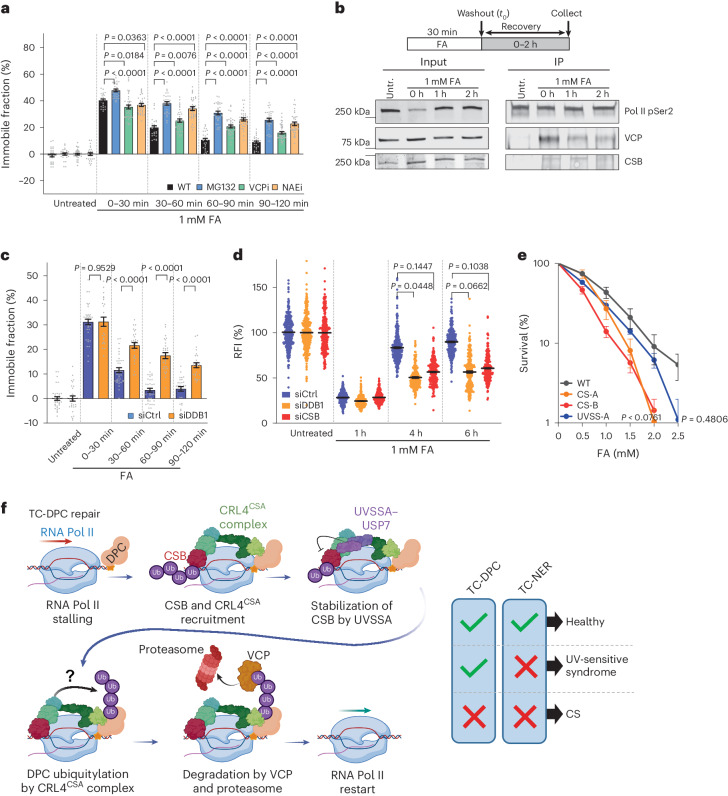
CRL4^CSA^ and proteasome-mediated removal of DPCs. **a**, Relative immobile fractions of non-replicating mScarlet-I–CSB FRAP in cells arrested following treatment with 1 µM palbociclib for 24 h. Cells were mock treated or treated with 50 µM proteasome inhibitor (MG132), 10 µM VCP inhibitor (VCPi; NMS873) or 20 µM neddylation inhibitor (NAEi; MLN4924) before treatment with 1 mM FA for 30 min. Values represent the mean ± s.e.m. *n* (left to right) = 25, 24, 34, 26, 24, 30, 40, 31, 27, 30, 39, 34, 27, 30, 39, 34, 27, 30, 40 and 33 cells from 3 (NAEi) or 4 (VCPi) independent experiments. Unpaired two-tailed *t*-test. **b**, Top: scheme of experiment. Bottom: IP of pSer2-modified elongating Pol II followed by immunoblotting with the indicated antibodies either directly after a 30 min FA pulse (1 mM) or after recovery for the indicated time. This experiment was performed twice with similar results. Untr., untreated. **c**, Relative immobile fractions of non-replicating mScarlet-I–CSB FRAP in siRNA-transfected cells, which were arrested in G1 with 1 μM palbociclib for 24 h before treatment with 1 mM FA for 30 min. Graphs represent the mean ± s.e.m. *n* (left to right) = 36, 28, 38, 24, 35, 28, 36, 28, 29 and 28 cells from 3 independent experiments. Unpaired two-tailed *t*-test. **d**, Quantification of recovery of transcription in RPE1 cells transfected with the indicated siRNA. Cells were treated with a 30 min FA (1 mM) pulse and left to recover for the indicated times, including a 30 min pulse labelling with EU. RFI of EU were normalized to untreated levels and set to 100%. Black lines indicate the average integrated density ± s.e.m. *n* (left to right) = 688, 652, 331, 633, 611, 466, 726, 391, 265, 721, 418 and 313 cells from 2 (siCSB) or 3 (siCtrl and siDDB1) independent experiments. Unpaired two-tailed *t*-test. **e**, Relative clonogenic survival assay in WT (MRC-5 sv40), CS-A (CS3BE sv40), CS-B (CS1AN sv40) and UVSS-A (TA-24 sv40) cells with the indicated doses of FA for a 1 h pulse. Graphs were normalized to the untreated colony number, which was set at 100%. Graphs represent the mean ± s.e.m. from five independent experiments. Unpaired two-tailed *t*-test. **f**, TC-DPC repair model. TC-DPC repair is initiated when DPC-stalled Pol II is recognized by CSB, which recruits CRL4^CSA^ E3 ligase. UVSSA stabilizes CSB through USP7. Ubiquitin (Ub)-DPC ubiquitylation by CRL4^CSA^ drives VCP-dependent and proteasome-dependent DPC degradation followed by transcription restart. Functional TC-DPC repair may explain the phenotypic differences in the TC-NER syndromes CS and UV-sensitive syndrome. Source numerical data (**a**,**c**–**e**) and unprocessed blots (**b**) are available in the source data. Scheme in **f** was created using BioRender (https://biorender.com). Source data

**Extended Data Fig. 10:**
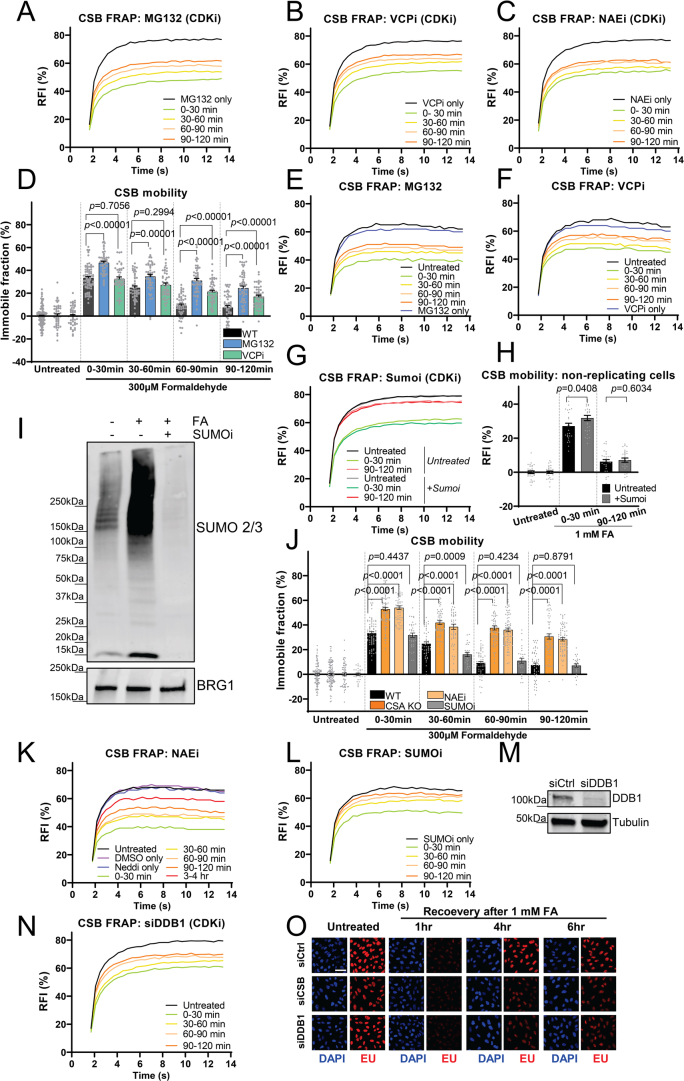
DPC repair required ubiquitination by CRL4^CSA^ and degradation by VCP-mediated proteasomal degradation. **a**: CSB-mScarlet-I FRAP with in non-replicating cells treated 1 μM Palbociclib (CDKi) for 24 hr prior to treatment with 50 µM proteasome inhibitor (MG132) and subsequently with 1 mM FA for 30 min. Relative immobile fraction as shown in Fig. 8a. Graph is an average of n = 24, 30, 30, 30, 30 cells (up to down) from 3 independent experiments. **b**: CSB-mScarlet-I FRAP with in non-replicating cells treated 1 μM Palbociclib (CDKi) for 24 hr prior to treatment with 10 µM VCP inhibitor (VCPi: NMS873) and subsequently with 1 mM FA for 30min. Relative immobile fraction as shown in Fig. 8a. Graph is an average of n = 34, 40, 39, 39, 40 cells (up to down) from 3 independent experiments. **c**: CSB-mScarlet-I FRAP with in non-replicating cells treated 1 μM Palbociclib (CDKi) for 24 hr prior to treatment with 20 µM neddylation inhibitor (NAEi: MLN4924) and subsequently with 1 mM FA for 30 min. Relative immobile fraction as shown in Fig. 8a. Graph is an average of n = 26, 31, 34, 34, 33 cells (up to down) from 3 independent experiments. **d**: Relative immobile fractions of mScarlet-I-CSB FRAP in HCT116 cells mock-treated, or treated with 50 µM MG132 and 10 µM VCP inhibitor (VCPi: NMS873) prior to treatment with 300 μM FA for 30 min. Values represent mean ± S.E.M., whereby 122, 50, 46, 82, 45, 48, 52, 48, 49, 56, 45, 52, 53, 51, 54 cells respectively (left to right) from 3 independent experiments were analyzed. Unpaired two-tailed t-test. **e**: CSB-mScarlet-I FRAP in HCT116 cells treated with 50 µM proteasome inhibitor MG132 prior to treatment with 300 μM FA for 30 min. Relative immobile fraction as shown in (**d**). Graph is an average of n = 46, 46, 48, 49, 52, 54 cells (up to down) from 3 independent experiments. **f**: CSB-mScarlet-I FRAP in HCT116 cells treated with 10 µM VCP inhibitor NMS873 (VCPi) prior to treatment with 300 μM FA for 30 min. Relative immobile fraction as shown in (**d**). Graph is an average of n = 38, 50, 45, 48, 45, 51 cells (up to down) from 3 independent experiments. **g**: CSB-mScarlet-I FRAP in non-replicating RPE1 cells treated 1 μM Palbociclib (CDKi) for 24 hr prior to treatment with 2 μM SUMO inhibitor (SUMOi: ML792) and subsequently with 1 mM FA for 30 min. Graph is an average of n = 29, 28, 27, 28, 28, 27 cells (up to down) from 3 independent experiments. **h**: Relative immobile fractions of mScarlet-I-CSB FRAP in non-cycling RPE1 cells mock treated, or treated with 2 μM SUMO inhibitor (SUMOi: ML792) prior to treatment with 1 mM FA for 30 min quantified from (**h**). Values represent mean ± S.E.M. Unpaired two-tailed t-test. **i**: Immunostaining with the indicated antibodies of chromatin fraction after formaldehyde treatment. Cells were pre-treated for 2 hr pretreatment with 2 μM sumoylation inhibitor (SUMOi) before treatment with 1 mM FA for 30 min. Cells were harvested immediately after FA treatment for chromatin fractionation. This experiment was performed twice with similar results. **j**: Relative immobile fractions of mScarlet-I-CSB FRAP in WT and CSA KO HCT116 cells and WT cells treated with 20 µM neddylation inhibitor (NAEi: MLN4924) or 2 μM SUMO inhibitor (SUMOi: ML792) prior to treatment with 300 μM FA for 30 min. Values represent mean ± S.E.M., whereby 122, 129, 61, 25, 82, 96, 57, 37, 52, 67, 49, 26, 56, 68, 74, 30, 53, 57, 65, 27 cells respectively (left to right) from a 3 (NAEi and Sumoi) or 4 (CSA KO) independent experiments were analyzed. Unpaired two-tailed t-test. **k**: CSB-mScarlet-I FRAP in HCT116 cells treated with 20 µM neddylation inhibitor (NAEi: MLN4924) prior to treatment with 300 μM FA for 30 min. Relative immobile fraction as shown in (**j**). Graph is an average of n = 66, 56, 61, 57, 49, 74, 65 cells (up to down) from 3 independent experiments. **l**: CSB-mScarlet-I FRAP in HCT116 cells treated with 2 μM SUMO inhibitor (SUMOi: ML792) prior to treatment with 300 μM FA for 30 min. Relative immobile fraction as shown in **(j)**. Graph is an average of n = 25, 37, 26, 30, 27 cells (up to down) from 3 independent experiments. **m**: Immunoblot of cells transfected with siRNA against DDB1, stained with antibodies against DDB1 and Tubulin. This experiment was performed twice with similar results. **n**: CSB-mScarlet-I FRAP in cells transfected with siRNAs against DDB1. Cells were treated with 1 μM Palbociclib (CDKi) for 24 hr prior to FRAP after 1 mM FA for 30 min. Relative immobile fraction as shown in Fig. 8c. Graph is an average of n = 28, 24, 28, 28, 28 cells (up to down) from 3 independent experiments. **o**: Representative pictures of the recovery of transcription in siRNA transfected RPE1 cells. Quantification is shown in Fig. 8d. Scale bar = 50 μm. Source numerical data and unprocessed blots are available in source data. Source data

Ubiquitylation of DPCs and their subsequent proteasomal degradation, mediated by the ubiquitin selective segregase VCP (also called p97), has been shown to play a major role both in replication-dependent and in global-genome DPC repair^7,10,61–64^. To test its involvement in TC-DPC repair, we studied FA-induced CSB immobilization over time in non-cycling cells to exclude indirect effects of the replication-dependent DPC repair. Both proteasome inhibition with MG132 and VCP inhibition with NMS873 resulted in severely reduced recovery of FA-induced CSB immobilization (Fig. 8a and Extended Data Fig. 10a–c), a result indicative for impeded TC-DPC repair. Similar results were obtained in cycling cells (Extended Data Fig. 10d–f). The involvement of VCP in TC-DPC repair was further corroborated by VCP recruitment to DPC-stalled Pol II (Figs. 3a,b and 8b). Together, these results indicate that the VCP-mediated proteasomal degradation of DPCs is crucial to overcome DPC-induced transcription stress.

DPC ubiquitylation by RNF4 depends on SUMOylation^7,10,57^. However, inhibition of SUMOylation did not affect the recovery of CSB mobility after FA exposure, which indicated that RNF4 is not involved in TC-DPC repair (Extended Data Fig. 10g–l). A logical candidate to ubiquitylate transcription-blocking DPCs, to instigate their degradation, is the CRL4^CSA^ ubiquitin ligase. Notably, neddylation inhibition, which prevents the activation of cullin-based ubiquitin ligases^65^, induced a similar delayed recovery of CSB mobility as that observed in *CSA* KO cells, which indicated that the ubiquitin ligase activity of the CRL4^CSA^ complex specifically is required for TC-DPC repair (Fig. 8a and Extended Data Fig. 10c,j–l). As neddylation inhibition affects all cullin-based ubiquitin ligases, we depleted DDB1, a crucial component of CRL4 ubiquitin ligase complexes^66^. DDB1 depletion resulted in a similar defect in CSB remobilization and transcription recovery as observed following CSB depletion (Fig. 8c,d and Extended Data Fig. 10m–o), which suggested that the activity of the CRL4^CSA^ complex is required for DPC ubiquitylation and its subsequent proteasomal degradation.

## Discussion

In this study, we showed that transcription is robustly inhibited by DPCs. However, transcription swiftly recovers, faster than the repair of total cellular DPCs^40^. This swift recovery is explained by the preferential repair of DPCs in transcribed genes compared with non-transcribed parts of the genome, which indicates that DPCs are repaired in a transcription-coupled manner, similar to that observed by other research groups^67,68^.

Notably, although recognition of lesion-stalled Pol II by the recruitment of CSB and CRL4^CSA^ is similar in TC-NER and TC-DPC repair, the mechanism to remove the transcription-blocking damage is markedly different (Fig. 8f). For TC-DPC repair, only CSB and CSA are essential, whereas more downstream factors such as UVSSA and XPA are not required. This has major consequences for the subsequent repair mechanism. In TC-NER, UVSSA is crucial for the binding of TFIIH to lesion-stalled Pol II, which, together with XPA, proofreads the lesion, followed by damage excision by the endonucleases XPG and ERCC1–XPF^18^. By contrast, during TC-DPC repair, UVSSA and XPA are dispensable, which indicates that the proofreading and damage excision steps are not part of this repair process. Moreover, although we observed only mild DPC sensitivity in *UVSSA* KO cells, in other studies, the role of UVSSA in TC-DPC repair seems more pronounced^67,68^. This difference could be explained by differences in the concentration or length of FA exposure, which could induce a different ratio of DPCs versus intrastrand and interstrand crosslinks. Alternatively, as UVSSA stabilizes CSB by recruiting USP7 (ref. ^24^), increasing levels of DPC-induced transcription stress will result in more CSB degradation, which could explain the larger contribution of UVSSA at higher DPC levels. Of note, at low endogenously induced DPC levels, for example, due to depletion of either alcohol or aldehyde dehydrogenases, UVSSA hardly affects survival in *C.* *elegans* (Fig. 4f), which indicates that at these more physiologically relevant DPC levels, UVSSA is not substanstially involved in TC-DPC repair.

An interesting question that is raised by these findings is why such a non-canonical TC-NER reaction has evolved. An obvious explanation may be that DPCs are much bigger than, for example, UV-induced lesions, which are repaired by TC-NER. TFIIH is expected to bind to the TC-NER complex on the downstream side of Pol II^18,20^, where the DPC is also located on the DNA. The bulky DPC might block the correct loading or the proofreading function of TFIIH and XPA owing to steric hindrance. As CSB and CSA bind on the upstream side of Pol II^20^, these factors are expected to bind DPC-stalled Pol II without any steric restraints.

We propose a model whereby the CRL4^CSA^ ubiquitin ligase complex is recruited to lesion-stalled Pol II to ubiquitylate the DPC. This ubiquitin ligase is important for ubiquitylation of both CSB and Pol II at K1268 of RPB1 (refs. ^20,22,23^), which are located at almost opposite sides of the TC-NER complex. This result indicates that the CUL4A and RBX1 components of the CRL4^CSA^ complex are highly flexible and can cover large molecular distances^20,69^. As the DPC would be located upstream of Pol II, relatively close to K1268 of RPB1, we postulate that the DPC itself could be a substrate of CRL4^CSA^. Our data suggest that following DPC ubiquitylation by CRL4^CSA^, the DPC is degraded by the 26S proteasome in a VCP-dependent manner. Notably, DPC ubiquitylation and subsequent proteasome-mediated degradation is a more common observed DPC clearance mechanism^3,61^. DPCs can by ubiquitylated by replisome-associated ubiquitin ligases^8^ or by the SUMO-targeted ubiquitin ligase RNF4 independent of replication^7,10^.

DPC degradation by the proteasome will significantly reduce the size of DPCs to much smaller peptide remnants. Although such small peptide remnants can be bypassed by the error-prone translesion synthesis DNA polymerases^70^, it is currently unknown whether such peptide remnants can be bypassed by Pol II. However, it has been shown that phage T7 RNA polymerase is able to transcribe over short DNA–peptide crosslinks, which could result in transcription errors^12^.

It is notable that mutations in TC-NER factors have been linked to two syndromes in humans with diverse phenotypes. Mutations in *CSA* and *CSB* cause severe Cockayne syndrome (CS), which is characterized by premature ageing and progressive neurodegeneration, whereas *UVSSA* mutations cause the mild UV-sensitive syndrome, with specific cutaneous phenotypes such as freckling and photosensitivity^18,24,71^. Cells from patients with CS and from patients with UV-sensitive syndrome are fully deficient in TC-NER and, as a consequence, equally sensitive to UV-induced DNA damage^24^. Here we showed that *CSA* and *CSB* KO cells are hypersensitive to DPC-induced transcription stress, whereas *UVSSA* KO cells showed only a mild sensitivity. This correlation between FA sensitivity and TC-NER disorder severity was further confirmed by the strong FA hypersensitivity of cells from patients with CS (CS-A and CS-B; Fig. 8e). By contrast, cells from patients with UV-sensitive syndrome had FA sensitivity levels near to that of WT cells (Fig. 8e). *C.* *elegans* mutants deficient in CSA and CSB demonstrate developmental arrest after depletion of aldehyde or alcohol dehydrogenases (Fig. 4f). This result suggests that DPCs formed by endogenous chemicals such as aldehydes may be an important driver of the premature ageing and severe neurodegeneration features observed in CS. In line with this hypothesis, it has been shown that increased aldehyde levels due to mutations in both *ALDH2* and *ADH5* result in aplastic anaemia, mental retardation and dwarfism (AMed) syndrome that is characterized by neuronal clinical features that overlap with CS^52,72^. Furthermore, mice deficient in ADH5 and CSB develop neurodegeneration and present other features that resemble CS^73^. FA induces interstrand and intrastrand DNA crosslinks in addition to DPCs^48^. However, *CSA* and *CSB* KO cells were also hypersensitive to 5-Aza-dC, whereas *UVSSA* and *XPA* KO cells were not (Fig. 4c). As 5-Aza-dC only induces enzymatic DPCs and no DNA–DNA crosslinks, this result indicates that DPCs are most likely to be the cause of these CS-like phenotypes. However, these aldehyde-induced DNA–DNA crosslinks could explain the minimal TFIIH recruitment to Pol II following FA exposure and the mild FA-induced sensitivity in *uvs-1* and *xpa-1 C.* *elegans* mutants.

TC-DPC repair is initiated by Pol II stalling, which therefore explains why DPCs can be repaired without the need for a specialized repair enzyme and irrespective of the identity of the crosslinked protein and varying size and structure. As transcription happens in both cycling and non-cycling cells, TC-DPC repair will be active irrespective of the cell cycle phase and will be specifically focused on the important transcribed part of the genome. By contrast, DPC recognition by the replisome or by SPRTN, which is expressed at low levels in G1 cells^7,58^, will probably not be efficient in non-replicating tissues. This might explain why severe CS phenotypes are particularly observed in post-mitotic cells such neurons, in which replication-dependent or genome-wide DPC repair pathways cannot act as a backup repair mechanism.

## Methods

### Cell lines and cell culture

All cells were cultured in a 1:1 mixture of Ham’s F10 and DMEM (Gibco), supplemented with 1% penicillin–streptomycin and 10% FBS (Capricorn Scientific) at 37 °C and 5% CO_2_ in a humidified incubator. siRNA transfections were performed 2 days before each experiment using Lipofectamine RNAiMax (Invitrogen) according to the manufacturer’s protocol. The following siRNAs were purchased from Horizon Discovery: siSPRTN: 5′-CACGAUGAGGUGGAUGAGUAU-3′ (ref. ^74^); siDDB1: 5′-UGAUAAUGGUGUUGUGUUU-3′; siCSA: 5′-CAGACAAUCUUAUUACACA-3′; siCSB: 5′-GCAUGUGUCUUACGAGAUA-3′; siUVSSA: 5′-GCUCGUGGAUCCAGCGCUU-3′; siUSP7: 5′-GCAUAGUGAUAAACCUGU AUU-3′; and siCtrl: 5′-UGGUUUACAUGUCGACUAA-3′.

The RPE1 CSB–mScarlet-I KI cell line was generated using the same strategy as previously described^34^. The GFP–DNMT1 construct was a gift from H. Leonhardt^75^ (Ludwig-Maximilians-Universität München), which was subcloned into a lentiviral vector and transduced into hTert-RPE1 cells. GFP-positive cells selected using 10 μg ml^–1^ blasticidin (Invivogen) and high expressing cells were sorted by FACS. To generate hTert-RPE1 KO cells, 100 pmol crRNA (Integrated DNA Technologies (IDT); Supplementary Table 2) was annealed with 100 pmol traRNA (IDT) in duplex buffer (IDT) by denaturing the oligonucleotides at 95 °C for 5 min and incubating at room temperature for 10 min. Subsequently, 6.5 µg Alt-R S.p. Cas9 nuclease V3 (IDT) was added to enable ribonucleoprotein (RNP) complexes to form. The RNP complexes were transfected with Lipofectamine CRISPRMAX Cas9 transfection reagent (Invitrogen) according to the manufacturer’s instructions. Clones were screened by PCR with MyTaq (Bioline) (Supplementary Table 2) and KOs were confirmed by TIDE analysis and immunoblotting. The SPRTN clone is heterozygous as the protein is essential for cell proliferation^76^.

Homozygous GFP–RPB1 KI MRC-5 human lung fibroblasts (SV40-immortalized) and the isogenic *CSA*, *CSB*, *XPA* and *XPC* KOs in this cell line have been previously described^33,29^. *UVSSA* KO cells were generated using a dual, doxycycline-inducible CRISPR–Cas9 vector system (iKRUNC, crRNA sequence: 5′-AGACACGAATGCTCGGAGTC-3′), and *UVSSA* KO in a single cell clone was verified by TIDE analysis and sequencing of a subcloned PCR fragment of the genomic targeting locus (forward primer: 5′-CATTCTCCTGCCTCAATCTC-3′; reverse primer: 5′-CCTGTGCCTGGCATCTCTG-3′). After obtaining this *UVSSA* KO clone, the *RPB1* locus was targeted with GFP as previously described^33^.

HeLa cells (WT, RPB1(K1268R) and *CSB* KO) were provided by the T. Ogi Laboratory^23^. The U2OS WT and *USP7* KO cells were shared by the Verrijzer Laboratory^56^. The *CSB* KO cells were generated by transfecting U2OS cells with Cas9 protein and the sgRNA targeting *CSB* sequence 5′-CTTCTCCACGTCAACGAGCT-3′ (IDT) using CRISPRMax Lipofectamine (Invitrogen). A *CSB* KO clonal cell line was isolated and verified by PCR genotyping, sequencing and immunoblotting.

HCT116 CSB–mScarlet-I KI cells have been previously described^34,46^. The ATPase mutant CSB HCT116 cell line was established through Nucleofection using 4D Nucleofector (Lonza, V4XC-1024) with purified Cas9 protein, traRNA, crRNA targeting 5′-CAAGAAGGCAATTATCTGGA-3′ and ssODN 5′- CCAGCAGGCAGGAGGAATTCTGGGAGATGAAATGGG ATTGGGCAGGACGATCCAGATAATTGCCTTCTTGGCAGGTCTGAGCTACAGCA-3′ (all ordered from IDT) following the manufacturer’s recommended protocol. Cells were grown in medium containing 2 nM of the DNA-PK inhibitor NU7441 (Selleckchem) and 10 μM of the Polθ inhibitor ART558 (MedChemExpress) for 2 days and subsequently grown in regular medium. Cell populations were then clonally expanded, and clones were screened by PCR and sequencing. For generating cells with mutations in the UBD of CSB, HCT116 cells were co-transfected with LentiCRISPR-V2 puro plasmid encoding a sgRNA (5′-AGAACACGATGACCTTCTGG-3′) targeting the UBD of *CSB* in exon 21, and with a designed ssODN (IDT) of 80 nucleotides containing the L1427G and L1428G mutations, as well as a modified PAM sequence, which does not affect the amino acid sequence (5′-AGTGTGGGCCTGGAAAGCGATGAAGTTTCTCATCTCGACCCCACCGTCATCGTGTTCTGTGGTGGGCAGCAGGGCAGAAG-3′). Cells were co-transfected using the jetPEI (PolyPlus) protocol according to the manufacturer’s instructions. Two days after transfection, cells were selected with 2 μg ml^–1^ puromycin, followed by single clone isolation, which were screened by PCR and sequencing. CS-A (CS3BE sv40), CS-B (CS1AN sv40) and UVSS-A (TA-24 sv40) cells have been previously described^24^.

The WTC-11 human iPS cell line (GM25256) containing eGFP–POLR2A (AICS096-074, Allen Cell Collection, Coriell Institute) was cultured in StemFlex medium (ThermoFisher Scientific, A3349401) on 0.08 mg ml^–1^ Geltrex, LDEV-Free, hESC-qualified, reduced growth factor basement membrane matrix (ThermoFisher Scientific, A1413301)-coated plates. Cells were kept at 37 °C, 5% CO_2_ and 3% O_2_, and were passaged in clumps by incubating with 0.5 mM EDTA.

*CSB* KO human iPS cell lines were obtained through RNP nucleofection using a Human Stem Cell Nucleofector kit 2 (Lonza, VPH-5022). Single cells were obtained through incubation in StemPro Accutase Cell Dissociation reagent (ThermoFisher Scientific) and nucleofected with RNPs containing Alt-R S.P. HiFi Cas9 nuclease V3 (IDT), Alt-R CRISPR–Cas9 tracrRNA, ATTO 550 (IDT) and target-specific Alt-R CRISPR–Cas9 crRNA (5′-CAAGAAGGCAATTATCTGGA-3′). Nucleofected cells were seeded on a plate in StemFlex medium with RevitaCell supplement (ThermoFisher Scientific). After 24 h, cells were subjected to FACS for ATTO 550, and positive cells were seeded 100–200 cells per cm^2^ in StemFlex medium with RevitaCell supplement and grown until picking of single-cell colonies. The presence of insertions and deletions was verified by PCR and Sanger sequencing of the targeted site.

Stable rtTA/NGN2 eGFP–POLR2A human iPS cell lines were generated by lentiviral transduction as previously described^60^. In brief, transduced cells were selected for 7 days with puromycin (Sigma) and G418 (Sigma). After selection, cells are maintained in Stemflex supplemented with puromycin (0.5 µg ml^–1^) and G418 (50 µg ml^–1^) and routinely passaged as single cells using Accutase until stored in liquid nitrogen. Human iPS cells were differentiated into excitatory cortical neurons through the overexpression of neurogenin-2 (NGN2) following the addition of doxycycline^59^. rtTA/NGN2-integrated human iPS cells were plated on coverslips pre-coated with 50 µg ml^–1^ poly-l-ornithine (Sigma), followed by coating with a 75 µl droplet of 80 µg ml^–1^ Matrigel (Corning). Next, 40,000 cells were seeded in a droplet of 75 µl seeding medium containing StemFlex medium with RevitaCell supplement and 4 µg ml^–1^ doxycycline (Sigma). After cell attachment, the well was filled with seeding medium. After 24 h, the medium was changed to DMEM/F12 (Gibco) supplemented with 1% N2 supplement (Gibco), 1% MEM non-essential amino acid solution (Gibco), 1% penicillin–streptomycin (Sigma Aldrich), 4 µg ml^–1^ doxycycline, 10 ng ml^–1^ human recombinant NT3 (StemCell Technologies), 10 ng ml^–1^ brain-derived neurotrophic factor (Prospec) and 0.2 µg ml^–1^ mouse laminin (Sigma). From day 3 onwards, cells were cultured in neurobasal medium (Gibco, 21103-049) containing 1% B27 supplement (Gibco), 1% Glutamax (Gibco), 1% penicillin–streptomycin, 4 µg ml^–1^ doxycycline, 10 ng ml^–1^ NT3, 10 ng ml^–1^ brain-derived neurotrophic factor and 2 µM cytosine β-d-arabinofuranoside (Sigma). All cultures were kept at 37 °C, 5% CO_2_ and 3% O_2_ throughout differentiation. FRAP experiments were performed between day 8 and day 12 of differentiation. Cytosine β-d-arabinofuranoside was removed from cultures at least 24 h before the FRAP experiments.

### Treatment with DNA-damaging agents and inhibitors

For FA treatment, a fresh vial of 16% (w/v) methanol-free FA (Pierce, 28906) was opened for every experiment. After a pulse of 30 min or 1 h (only when used for survival assays) at the indicated doses, cells were washed 3 times with DMEM/F10 (1:1) medium with 10% FBS to wash away and quench the FA. The cells were subsequently incubated as described above until collection or imaging. To generate DNMT1 DPCs for transcription analysis, cells at 50–70% confluency were incubated for 30 min with 50 μM 5-Aza-dC (Sigma, A3656) and subsequently cultured in standard culture medium until transcription was assayed as described below. For survival assays, 5-Aza-dC was added to the medium at the indicated concentrations and left on the cells until fixation. For UV irradiation, cells were washed with PBS and placed under a 254 nm germicidal UV-C lamp (Philips), whereby the duration of irradiation was controlled with an air-pressured shutter connected to a timer to expose cells to the indicated UV dose. For analysis of Pol I and Pol II transcription, cells were treated with 50 ng ml^–1^ actinomycin D (Sigma) or 1 μM flavopiridol (Sigma) for 1 h before transcription analysis. Cells were treated with the VCP inhibitor NMS873 (10 µM, Selleck Chemicals) directly together with FA treatment. Cells were pre-treated for 30 min with the NEDD8 E1 activating enzyme inhibitor MLN4924 (20 µM, Boston Biochem) or for 1 h with 50 µM MG132 (Enzo) or the CDK7 inhibitor THZ1 (1 µM for recovery of RNA synthesis and CSB FRAP and 2 µM for RPB1 FRAP and TCR-UDS). Cells were pre-treated with the SUMO inhibitor ML792 (2 μM, SelleckChem) for 30 min before FA before FRAP experiments and 2 h before fractionation. Cells were arrested in G1 through treatment with 1 µM of the CDK4 and CDK6 inhibitor palbociclib for 24 h before assays.

### *C.elegans* strains and methods

*C.* *elegans* were cultured according to standard methods on nematode growth medium agar plates seeded with *Escherichia coli* OP50. *C.* *elegans* strains used were WT (Bristol N2), *xpa-1*(*ok698*)^77^, *csa-1*(*tm5232*)^50^, *csb-1*(*emc79*) and *uvs-1*(*emc80*). Animals with complete removal of *csb-1* (designated *emc79*) or *uvs-1* (designated *emc80*) were generated by injection of WT animals with Alt-R S.p. Cas9 nuclease V3 (IDT) and sgRNAs targeting *csb-1* (5′-TGAAAAAATACCTAAGTACC-3′ and 5′-AAAAATGAATCAATGAATAA-3′) or *uvs-1* (5′-CAAATAAAATGTTGAAAAGA-3′ and 5′-CAGTTTTCTCATTTTTAATA-3′). Mutant animals were selected and verified by genotyping PCR and sequencing (Extended Data Fig. 3e,f). RNAi bacteria to deplete *alh-1* and *adh-5* were obtained from the *C.* *elegans* RNAi feeding library^78^. Control RNAi was the vector L4440 (Addgene, plasmid 1654; a gift from A. Fire). RNAi depletion was achieved by culturing animals for three generations on RNAi bacteria before the start of each experiment. For each RNAi growth experiment, young adults grown on RNAi bacteria were allowed to lay eggs on five 6 cm RNAi plates for 4 h. Four days later, developmental growth was scored by counting the number of eggs, different larval stages and adult animals on the plate. For each FA survival experiment, staged young adults were allowed to lay eggs for 24 h on four 6 cm plates containing the indicated dose of FA. After 24 h, survival was scored by counting dead and living offspring. UV survival assays were performed according to the L1 larvae UV survival assay as previously described^79^. In brief, *E.* *coli* OP50, transformed with RNAi, was spread on LB agar plates, and young adult worms were allowed to lay eggs for 4 h. Four days later, the developmental stages of the offspring were counted.

### Clonogenic survival assay

For survival assays, 300 cells (RPE1 and MRC-5), 500 cells (HeLa, HCT116, CS1AN, CS3BE and TA24 cells) or 600 cells (U2OS) were seeded per well in triplicate in a 6-well plate. The following day, cells were treated with DNA-damaging agents. For FA, the cells were treated for 1 h and subsequently washed 3 times in culture medium. For 5′-Aza-dC (Sigma), the cells were treated with the indicated doses, which was left on the cells until fixation. For cisplatin, the cells were treated for 24 h and subsequently thoroughly washed with culture medium. After allowing the cells to grow in colonies for 7–10 days, the plates were fixed and stained using Coomassie blue (50% methanol, 7% acetic acid and 0.1% Coomassie blue (all Sigma)) and colony numbers were counted using GelCount (Oxford Optronix). The relative colony number was plotted from at least two independent experiments, each performed in triplicate. Levels were normalized to mock-treated, set to 100 and plotted with s.e.m. values.

To assess cell viability in non-replicating cells Alamar Blue (Invitrogen) survival assays were used, which uses a resazurin-based solution that functions as a cell health indicator by using the reducing power of living cells to quantitatively measure viability. RPE1 cells were seeded to confluency in triplicate in 96-well plates. The next day, cells were arrested in G1 with 1 µM palbociclib (SelleckChem), which remained present during the entire experiment. At 48 h after palbociclib treatment, cells were treated with FA at the indicated doses for 1 h and then washed 3 times with regular culture medium. To determine viability of the cells 96 h after FA treatment, metabolic activity was measured using Alamar Blue, which was added for 2 h, and the fluorescence was measured at 570 nm using a SpectraMax iD3 reader (Molecular devices). Data were normalized to mock-treated conditions.

### Cell lysis and immunoblotting

Cells were directly lysed in SDS–PAGE sample buffer (125 mM Tris pH 6.8, 2% SDS, 0.005% bromophenol blue, 21% glycerol and 4% β-mercaptoethanol). For chromatin-bound Pol II and SUMOylation and chromatin associated proteins, cells grown in a 6-well plate were lysed in fractionation buffer with 30 mM HEPES pH 7.5, 130 mM NaCl, 1 mM MgCl_2_, 0.5% Triton X-100 with 50 µM MG132, complete EDTA-free protease inhibitors (Roche), phosphatase inhibitor cocktail 2 (Sigma) and *N*-ethylmaleimide (Sigma) for 30 min on ice. Chromatin was pelleted at 15,000*g* for 10 min at 4 °C and washed once with fractionation buffer. Finally, the chromatin was digested with 50 U benzonase (Millipore) for 30 min at 4 °C before adding SDS–PAGE sample buffer followed by 5 min of incubation at 95 °C. Protein samples were separated on 4–15% Mini-Protean TGX precast protein gels (Bio-Rad) in 25 mM Tris, 192 mM glycine and 0.1% SDS buffer. Proteins were transferred onto polyvinylidene difluoride membranes (0.45 µm, Merck Millipore) at 4 °C overnight at 30 V in transfer buffer (25 mM Tris, 192 mM glycine and 10% ethanol or 25 mM Tris and 192 mM glycine for chromatin fractionations). Membranes were blocked with 3% BSA (Sigma) in PBS and probed with primary antibodies in 1% BSA in PBS. Subsequently, membranes were extensively washed with PBS with 0.05% Tween and incubated with secondary antibodies coupled to IRDyes (LI-COR) to visualize proteins using an Odyssey CLx infrared scanner (LI-COR). Image Studio Lite (v.5.2.5) was used for western blot acquisition and analysis.

The following primary antibodies were used: rabbit anti-BRG1 (Abcam, ab110641; 1:2,000); rabbit anti-CSA (Abcam, ab240096; 1:1,000); rabbit anti-CSB (Antibodies Online, ABIN2855858; 1:1,000); rabbit anti-DDB1 (Novus Biologicals, NBP2-75465; 1:1,000); rabbit anti-RPB1 pSer2 (Abcam, ab5095; 1:1,000) or rat anti-RPB1-pSer2 (Chromotek, 3E10; 1:1,000); rat anti-RPB1-phospho-Ser5 (Chromotek, 3E8; 1:1,000); mouse anti-SSRP1 (Biolegend, 609701; 1:10,000); rabbit anti-SPRTN (Invitrogen, PA5-46262; 1:500); mouse anti-SUMO2/3 (Proteintech, 67154-1-1g; 1:1,500); mouse anti-tubulin (Sigma-Aldrich, T5168, 1:5,000); rabbit anti-USP7 (Bethyl, A300-033A; 1:1,000); rabbit anti-VCP (Bethyl, A300-589A; 1:1,000); rabbit anti-XPA (Genetex, GTX103168; 1:1,000); rabbit anti-XPB (Abcam, ab190698; 1:1,000); and rabbit anti-XPC (Bethyl, A301-112A; 1:2,000). The following secondary antibodies were used: goat anti-rabbit conjugated to IRdye (Sigma, SAB4600215 (770) or SAB4600200 (680); both 1:10,000); goat anti-mouse conjugated to IRdye (Sigma, SAB4600214 (770) or SAB4600199 (680); both 1:10,000); and goat anti-rat conjugated to IRdye770 (Sigma, SAB4600479; 1:10,000).

### FRAP analysis

CSB, RPB1 and DDB2 FRAP analyses were performed as previously described^29,33,34,80^. In brief, for RPB1 and DDB2 FRAP, a Leica TCS SP5 microscope (LAS AF software, v.2.7.4.10100, Leica) with a HCX PL APO CS ×63, 1.40 NA oil-immersion lens or, for CSB FRAP, a Leica TCS SP8 microscope (LAS X software, v.3.5.6.21594, Leica) with a HC PL APO CS2 ×63, 1.40 NA oil-immersion lens was used. Cells were maintained at 37 °C and at 5% CO_2_ during imaging. Two narrow strips, one inside and another outside the nucleus, of 512 × 32 pixels (RPB1 and DDB2) or 512 × 16 pixels (CSB) were imaged every 400 ms at 400 Hz using a 488 nm laser (RPB1), every 800 ms at 400 Hz using a 488 nm laser (neuronal RPB1), every 400 ms at 561-nm laser (CSB) or every 22 ms at 1,400 Hz (DDB2) using a 488 nm laser. A total of 25 (RPB1), 200 (DDB2) or 5 (CSB) frames were measured to reach steady-state levels before photobleaching (1 frame 100% laser power for RPB1, 2 frames for CSB and 7 frames for DDB2). After photobleaching, the recovery of fluorescence was measured using 450 (RPB1) 300 (neuronal RPB1), 30 (CSB) or 1,500 (DDB2) frames. Relative fluorescence intensity (RFI) was corrected for the background signal quantified outside the nucleus and normalized to the average pre-bleach fluorescence intensities. Immobile fractions were calculated according to the following formula:

$${\rm{Immobile}}\; {\rm{fraction}}=1-\frac{({\rm{I}}\; {\rm{recovery}},{\rm{FA}}- < {\rm{I}}\; {\rm{bleach}} > )}{( < {\rm{I}}\; {\rm{recovery}},{\rm{NT}} > - < {\rm{I}}\; {\rm{bleach}} > )}$$

whereby I recovery, FA refers to the average recovery of fluorescence of frames 414–450 (RPB1) or frames 16–30 (CSB). This was normalized to the average recovery of all untreated cells (<I recovery, NT>) and the average first measurement after bleaching (<I bleach>).

### Pol II IP

Elongating Pol II pSer2 was immunoprecipitated as previously described^34^. In brief, cells on 3 confluent 15 cm dishes for IP followed by immunoblotting or 10 confluent 15 cm dishes for SILAC interaction proteomics were treated with FA or UV as indicated. After collection by trypsinization, cells were collected in cold PBS and centrifuged for 5 min at 438*g* at 4 °C. After 2 cold PBS washes, cell pellets were stored at −80 °C until IP.

For IP, cell pellets were thawed on ice and lysed in buffer B1 (30 mM HEPES pH 7.6, 1 mM MgCl_2_, 150 mM NaCl and 0.5% NP-40 with 1× complete EDTA-free protease inhibitor cocktail (Roche)) and rotated for 20 min at 4 °C. Chromatin was pelleted by centrifuging at 10,000*g* for 5 min at 4 °C. After a wash in buffer B1, the chromatin was digested in buffer B1 containing 500 U benzonase (Millipore) and incubated with 2 µg RPB1 pSer2 antibody (Abcam, ab5095) for 1 h at 4 °C. After 1 h, the NaCl concentration was increased to 300 mM to remove weak interactors and to inactivate benzonase and incubated for an additional 30 min. After spinning at 16,800*g* for 10 min at 4 °C, the soluble supernatant containing the antibody-bound fraction was incubated with 25 µl slurry salmon sperm protein A agarose beads (Millipore) for 2 h while rotating at 4 °C. Unbound proteins were removed by washing the beads 5 times in buffer B2 (30 mM HEPES pH 7.6, 150 mM NaCl, 1 mM EDTA and 0.5% NP-40 with 0.2× complete EDTA-free protease inhibitor cocktail). Bound elongating Pol II complexes were eluted off the beads in SDS–PAGE sample buffer and separated on 4–15% Mini-Protean TGX precast protein gels (Bio-Rad). Samples were transferred to polyvinylidene difluoride membranes for immunoblotting or fixed and stained for mass spectrometry using Imperial protein stain (Pierce) according to the manufacturer’s protocol.

### SILAC–mass spectrometry

For SILAC, cells were grown for 2 weeks (>10 cell doublings) in arginine/lysine-free SILAC DMEM (ThermoFisher) supplemented with 10% dialysed FCS (Gibco), 1% penicillin–streptomycin, 200 µg ml^–1^ proline (Sigma) and either 73 μg ml^–1^ light [^12^C6]-lysine and 42 μg ml^–1^ [^12^C6,^14^N4]-arginine (Sigma) or heavy [^13^C6]-lysine and [^13^C6,^15^N4]-arginine (Cambridge Isotope Laboratories). Pol II was immunoprecipitated from the cells as described above.

### Mass spectrometry analysis

SDS–PAGE gel lanes were cut into slices and subjected to in-gel reduction with dithiothreitol (Sigma, D8255), alkylation with iodoacetamide (Sigma, I6125) and digestion with trypsin (sequencing grade; Promega) as previously described^34^. Nanoflow liquid chromatography–tandem mass spectrometry was performed on an EASY-nLC 1200 coupled to a Lumos Tribid Orbitrap mass spectrometer (ThermoFisher Scientific) operating in positive mode. Peptide mixtures were trapped on a 2 cm × 100 μm Pepmap C18 column (Thermo Fisher, 164564) and then separated on an in-house packed 50 cm × 75 μm capillary column with 1.9-μm Reprosil-Pur C18 beads (Dr. Maisch) at a flow rate of 250 nl min^–1^ using a linear gradient of 0–32% acetonitrile (in 0.1% formic acid) over 120 min. The eluate was directly sprayed into the electrospray ionization source of the mass spectrometer. Spectra were acquired in continuum mode, and fragmentation of the peptides was performed in data-dependent mode by HCD.

Mass spectrometry data were analysed using MaxQuant software (v.1.6.3.3). The false discovery rate of both peptide-spectrum match and protein was set to 0.01 and the minimum ratio count was set to 1. The Andromeda search engine was used to search the tandem mass spectrometry spectra against the UniProt database (taxonomy: *Homo sapiens*, release 2018), concatenated with the reversed sequences of all proteins. A maximum of two missed cleavages was allowed. In case the identified peptides of two proteins were the same or the identified peptides of one protein included all peptides of another protein, these proteins were combined using MaxQuant and reported as one protein group. Before further analysis, known contaminants and reverse hits were removed. GO term enrichment analysis, which included genes with an average SILAC log_2_ ratio of >1.2, was performed using the g:Profiler website^81^ to identify the top 10 biological processes and reactions after FA treatment.

### Recovery of RNA synthesis

Cells were grown on coverslips for 48 h before treatment with DNA-damaging agents as indicated. Transcription levels were measured by pulse labelling with 100 µM EU (Jena Bioscience) in regular culture medium, and cells were grown at 37 °C for 30 min before fixation with 3.6% FA (Sigma) in PBS for 10 min at room temperature. After permeabilization with 0.1% Triton X-100 in PBS for 10 min, Click-it-chemistry-based azide coupling was performed by incubating for 1 h with 60 µM Atto594 azide (Attotec) in 50 mM Tris buffer (pH 8) with 4 mM CuSO_4_ (Sigma) and 10 mM freshly prepared ascorbic acid (Sigma). Coverslips were washed with 0.1% Triton X-100 in PBS and with PBS only. For 5-Aza-dC-treated cells, coverslips were incubated with mouse anti-PCNA (Abcam, ab29, 1:200) and rabbit anti-DNMT1 (CST, 5032, 1:200) in 1% BSA–PBS for 1 h at room temperature. The coverslips were washed with PBS 3 times before staining with secondary antibodies conjugated with Alexa488 or Alexa633 (Invitrogen, 1:1,000) in 1% BSA–PBS for 30 min at room temperature.

Nuclei were visualized using 100 ng ml^–1^ 4,6-diamidino-2-phenylindole (DAPI; Brunschwieg Chemie). Coverslips were mounted with Aqua-Poly/Mount (Polysciences). Cells were imaged using a Zeiss LSM 700 Axio Imager Z2 upright microscope equipped with a ×40 Plan-apochromat 1.3 NA oil-immersion lens (Carl Zeiss Micro Imaging) using Carl Zeiss LSM software (v.14.0.0.0). The integrated density of the EU signal in the nuclei was quantified using ImageJ/Fiji software (v.1.52p) with a macro identifying the surface of each nucleus based on the DAPI signal, after which the mean fluorescence of the EU signal in the nucleus was measured. The mean fluorescence intensity was corrected for the background signal. With these values, the integrated density was calculated and plotted as single-cell points with the average and s.e.m.

For 5-Aza-dC-treated cells, the lines in ImageJ/Fiji were drawn across DNMT1 foci at a width of 5 pixels and the average of the 5 pixels is plotted. For quantifying transcription levels in foci and the surrounding nucleoplasm, the nucleus was segmented based on the DAPI signal and DNMT1-DPC foci and nucleoli were identified using Yen’s thresholding^82^. The nucleoli were subtracted from the foci area to avoid any overlap before obtaining the damage mask. To determine the global EU signal of the nucleoplasm, the foci and nucleoli were subtracted from the nucleus mask. The resulting masks were used to determine the mean fluorescence of the EU signal of the area of interest, foci or global, respectively. For global EU intensity measurement in control cells, the nucleoli were again excluded from the nuclear area. The macro used for segmenting foci to measure transcription in DNMT1 foci and in the nucleoplasm is available at GitHub (https://github.com/Marteijnlab/DPC-transcription-stress.git).

### TCR-UDS

The assay was performed as previously described^55^. In brief, XPC-deficient RPE1 cells, grown on glass coverslips, were arrested in G1 with the CDK inhibitor 1 µM palbociclib for 24 h. After a 30 min of treatment as indicated, cells were washed 3 times with medium before nascent DNA was labelled for 7 h with 20 µM EdU in Ham’s F10 medium supplemented with 1 µM palbociclib, 10% FCS, 1% penicillin–streptomycin and 1 µM floxouridine (Sigma) at 37 °C. After labelling, unincorporated EdU was removed by cold chasing the cells in Ham’s F10 supplemented with 1 µM palbociclib, 10% FCS and 1% penicillin–streptomycin with 10 µM thymidine (Sigma). Cells were fixed with 3.6% FA and 0.5% Triton-X 100 in PBS for 15 min at room temperature and permeabilized with 0.5% Triton for 20 min followed by blocking in 3% BSA at 4 °C overnight. After blocking, cells were washed in PBS, and endogenous peroxidase activity was quenched by incubating cells in 3% H_2_O_2_ (Sigma) in PBS for 30 min. Subsequently, cells were washed in PBS with 0.5% BSA and 0.15% glycine for 1 h and the Click-it chemistry reaction was performed using 200 µM PEG3–biotin–azide (Jena Bioscience), 1× Click-it reaction buffer, 4 mM CuSO_4_ solution and 10× reaction buffer additive (C10337, Thermo Fisher kit) for 1 h. The UDS signal was amplified using HRP–streptavidin (Thermo Fisher, B40932) for 1 h and the signal was visualized using Alexa-Fluor 488-nm-conjugated tyramide (B40932 kit) for 10 min, after which the reaction was stopped using HRP-Reaction stop reagent (from the B40932 kit) for 3 min, and cell nuclei were stained using DAPI (100 ng ml^–1^ in PBS) for 15 min. Coverslips were mounted on glass slides using AquaPoly/Mount.

### Quantitative flow cytometry

For quantitative flow cytometry, cells were seeded in a 6-well plate 2 days before treatment. Cells were washed in PBS and exposed to 1 mM FA in culture medium for 30 min at 37 °C. For the GFP–RPB1 levels, new protein synthesis was inhibited with 100 µM cycloheximide (Sigma) by pre-treating for 2 h before FA treatment and keeping it in the medium throughout the FA treatment and recovery period afterwards. The proteasome inhibitor MG132 (50 μM) was added together with the FA and remained present in the culture medium throughout the incubation period. Cells were then collected by trypsinization, centrifuged for 3 min at 281*g* and resuspended in 500 µl PBS containing 1% FA. Cells were analysed on a LSRFortessa X-20 Cell Analyzer (BD) equipped with FACSDiva software (BD). Cellular GFP–RPB1 or CSB–mScarlet-I protein levels were quantified after exclusion of dead cells by granularity (SSC-A) and size (FSC-A) using a 488 nm laser and 530/30 filter for GFP–RPB1 and using a 561 nm laser and 610/20 filter for CSB–mScarlet-I. Flow cytometry data were analysed using FlowJo software (v.10.8.1) from BD Biosciences. Fluorescence intensity was corrected and normalized to mock-treated fluorescence.

### Flavopiridol qPCR analysis

MRC-5 cells were grown in 6-well plates and treated for 2 h with 1 μM flavopiridol (Sigma) before 30 min of FA pulse (1 mM). Cells were washed 3 times with culturing medium before RNA was isolated. Cells were lysed on the plate and RNA was isolated using a RNeasy mini kit (Qiagen, 74104) following the manufacturer’s instructions. Genomic DNA was digested on the column with RNase free DNase (Qiagen, 79254). RNA concentrations were determined on a Nanodrop, and 1 μg RNA was used to make cDNA using an iScript cDNA synthesis kit (Bio-Rad, 170-8891). RT–qPCR was performed using 15 ng cDNA in triplicate with 3 µl DNA, 1 µl 5 μM primer mix and 6 µl 2× PowerUp SYBR Green master mix (Thermo Fisher, A25778) per reaction in 384-well plates on a CFX384 Touch Real-Time PCR system (Bio-Rad). Primers used are listed in Supplementary Table 3. DNA was amplified using the following program: 95 °C for 5 min, 40 cycles of 15 s at 95 °C and 50 s at 60 °C followed by a dissociation curve (from 65 °C to 95 °C with an increment of 0.5 °C for 5 s). Values were calculated using the 2-ΔCt method, whereby each condition was normalized to the 0 min sample associated with the treatment.

### Nascent RNA-seq

MRC-5 cells, grown to 80% confluency, were incubated with 1 mM of EU for 10 min followed by RNA isolation. Nascent RNA was isolated using a Click-iT Nascent RNA Capture kit (Thermo, C10365) per the manufacturer’s instructions at the maximum recommended input quantities. The beads with EU–RNA were then resuspended in the fragment, prime and elute buffer supplied with the KAPA RNA HyperPrep kit (Roche, KK8540). The mixture was heated to 94 °C for 6 min, and library preparation was completed according to the manufacturer’s instructions. The libraries were amplified with 15 PCR cycles, followed by clean-up. The quality and quantity of the library were assessed using a High Sensitivity D1000 assay on a TapeStation system (Agilent). Equal input quantities were then sequenced on a NovaSeq 6000 system (Agilent). EU sequencing reads were preprocessed using FastQC (v.0.11.9), FastQScreen (v.0.14.0) and Trimmomatic (v.0.35)^83^. The remaining reads were then aligned to the human ribosomal DNA, mitochondrial sequences (UCSC, hg38), and the human reference genome (GRCm38) using Tophat2 (v.2.0.9)^84^, with default settings (except for the -g 1 option).

### DPC removal assay

This protocol is described in more detail at Protocol Exchange^42^. MRC-5 cells were seeded on 6-well plates (DPC-qPCR) or 10 cm dishes (DPC-seq) and mock treated or exposed to 1 mM FA for 30 min. THZ (1 μM; SelleckChem) was added 1.5 h before FA treatment for the indicated samples. Cells were wash 2 times with PBS and lysed in 900 μl 2% SDS solution with 10 mM Tris-HCl (pH 7.5). Samples were stored at −20 °C after snap-freezing in liquid nitrogen until further processing. After thawing at 55 °C, DNA was sheared by passing the lysates through a 23-gauge needle 5 times and subsequent sonication at room temperature with high amplitude and 30 cycles of 30 s on and 30 s off (Bioruptor Plus, Diagenode, B01020001). Next, samples were incubated at 55 °C for 10 min. An equal volume (900 μl) of precipitation buffer (400 mM KCl and 20 mM Tris-HCl, pH 7.5) was added and samples were incubated at 4 °C for 6 min to complete precipitation. After centrifugation at 20,000*g* for 5 min at 4 °C, the supernatants were collected. The resulting pellets were washed at 55 °C for 10 min with 1 ml of wash buffer (200 mM KCl and 20 mM Tris-HCl, pH 7.5), incubated on ice for 6 min and centrifuged at 20,000*g* for 5 min at 4 °C. This washing procedure was repeated twice. All supernatants were combined for free DNA measurement. DPC-associated pellets were resuspended in 400 μl resuspension buffer (0.2 mg ml^–1^ proteinase K and 0.2 mg ml^–1^ RNAse A in 100 mM KCI, 20 mM Tris-HCl and 10 mM EDTA) by vortexing. The samples were then incubated at 50 °C. After 3 h of incubation, samples were chilled on ice for 6 min and centrifuged at 20,000*g* for 10 min at 4 °C to remove debris. The supernatant, which contains the DPC-associated DNA, was collected and purified using a gel extraction kit (Qiagen, 20051). DPC levels were determined by RT–qPCR and DNA sequencing.

RT–qPCR was performed on the eluted DNA in triplicate using 5 µl DNA, 1 µl primer mix and 7 µl 2× PowerUp SYBR Green master mix (Thermo Fisher, A25778) per reaction in 384-well plates on a CFX384 Touch Real-Time PCR system (Bio-Rad). Primers used are listed in Supplementary Table 4. DNA was amplified using the following program: 50 °C for 2 min, 95 °C for 2 min, 45 cycles of 15 s at 95 °C and 1 min at 58 °C followed by a dissociation curve (95 °C for 10 s and heating from 65 °C to 95 °C with an increment of 0.5 °C for 5 s each). Data collection was enabled at each increment of the dissociation curve. DPC-associated DNA levels were normalized to free DNA levels using the 2-ΔΔCt method, after which the FA-treated samples were normalized to the non-treated samples. Subsequently, all samples were normalized to the FA 0 h sample.

For DPC-seq, 50 ng DNA was used for the Twist Library Preparation EF kit 2.0. The fragmentation step was omitted and 7 PCR cycles were performed. Sequencing was done using a S1 flowcell on a Novaseq6000 with 2 × 150 cycles. All sequencing data were preprocessed using fastp (v.0.23.4) with its default options^85^. Data were aligned to the GENCODE GRCh38 release 44 reference genome^86^. RNA-seq data alignment used STAR (v.2.7.11a) with the alignment options ‘–runMode alignReads–outSAMunmapped Within–outSAMattributes Standard’^87^. DNA data were aligned using bwa (v.0.7.17-r1188) with the alignment option ‘mem’.

The genome was partitioned into bins of 1,000 bases each, and read counts for the bins were computed from every sequencing sample using featureCounts (v.2.0.6) with the counting options ‘-O–fraction -s 0 -p–countReadPairs -B–primary–ignoreDup -Qz30’^88^. All additional data processing was performed on the binned datasets. Replicates (where applicable) were combined by computing the average total reads for the combined sample sets, scaling bins in each sample to this average, then averaging the corresponding bins between the samples. Filtering of data was performed by discarding bins from all datasets (DNA and RNA) whenever a filter criterion was met by any bin in any of the datasets. The DNA outlier bins were discarded using the IQR method, computing quartiles and removing bins below Q1 – 1.5 × IQR and above Q3 + 1.5 × IQR. Q1 – 1.5 × IQR was below zero, so no low-end bins were discarded in this manner. Additionally, DNA bins with a TPM of less than 0.08 were discarded as they provided limited opportunity for repair and increased the noise in the data. After this filtering, 73.4% of bins remained. For comparisons between the 0 h and 4 h samples (after replicate combining), the RNA-seq bins with zero reads (indicating no transcription) were selected to use as a reference for data normalization. DNA bins corresponding to these zero transcription RNA bins were summed and a ratio was computed between samples. The ratio was then used to scale 0 h sample bins to the 4 h level. For the purposes of statistical analysis and plotting of the data, a final RNA TPM threshold filter was applied, removing all bins corresponding to RNA bins with TPM less than or equal to the threshold. Thresholds are specified with each graph or set of statistics. If no threshold is specified, then all bins with RNA TPM equal to zero were excluded.

### Statistics and reproducibility

Experimental data were analysed and processed in Excel (2016) and plotted using GraphPad Prism 9.4.0 (GraphPad Software) using unpaired two-tailed *t*-tests. The number of experiments, sample size and statistic tests are reported in the respective figure legends. Clonogenic survival assays, recovery of RNA synthesis, TC-UDS, FRAP experiments, DPC–qPCR, RT–qPCR and flow cytometry assays were performed three times unless specified in the legends. IP and western blotting characterizing cell lines were performed two times.

No statistical method was used to predetermine sample size. No data were excluded from the analyses. The experiments were not randomized. The investigators were not blinded to allocation during experiments and outcome assessment.

### Reporting summary

Further information on research design is available in the Nature Portfolio Reporting Summary linked to this article.

## Online content

Any methods, additional references, Nature Portfolio reporting summaries, source data, extended data, supplementary information, acknowledgements, peer review information; details of author contributions and competing interests; and statements of data and code availability are available at 10.1038/s41556-024-01394-y.

### Supplementary information

Reporting Summary

Supplementary Tables Supplementary Table 1: Table with SILAC ratios and peptide numbers as determined using quantitative interaction proteomics. Supplementary Table 2: List of crRNA sequences and sequencing primers for generating *RPE1* KO cell lines. Supplementary Table 3: List of primers used in real-time qPCR in flavopiridol qPCR assays. Supplementary Table 4: List of primers used in real-time qPCR in DPC assays.

### Source data

Source Data Fig. 1 Statistical source data.

Source Data Fig. 3 Statistical source data.

Source Data Fig. 3 Unprocessed western blots.

Source Data Fig. 4 Statistical source data.

Source Data Fig. 5 Statistical source data.

Source Data Fig. 6 Statistical source data.

Source Data Fig. 7 Statistical source data.

Source Data Fig. 7 Unprocessed western blots.

Source Data Fig. 8 Statistical source data.

Source Data Fig. 8 Unprocessed western blots.

Source Data Extended Data Fig. 1 Statistical source data.

Source Data Extended Data Fig. 1 Unprocessed western blots.

Source Data Extended Data Fig. 2 Statistical source data.

Source Data Extended Data Fig. 3 Statistical source data.

Source Data Extended Data Fig. 4 Statistical source data.

Source Data Extended Data Fig. 5 Unprocessed western blots.

Source Data Extended Data Fig. 6 Statistical source data.

Source Data Extended Data Fig. 7 Statistical source data.

Source Data Extended Data Fig. 7 Unprocessed western blots.

Source Data Extended Data Fig. 8 Statistical source data.

Source Data Extended Data Fig. 8 Unprocessed western blots.

Source Data Extended Data Fig. 9 Statistical source data.

Source Data Extended Data Fig. 10 Statistical source data.

Source Data Extended Data Fig. 10 Unprocessed western blots.

## Data Availability

SILAC-based Pol II quantitative interaction data have been deposited into the ProteomeXchange Consortium through the PRIDE partner repository with the dataset identifier PXD041679. Any other data are available from the corresponding author upon reasonable request. Nascent RNA-seq data are available under Sequence Read Archive (SRA) BioProject identifier PRJNA1017406 and Biosample identifiers SAMN37395210 and SAMN37395212. DPC-seq data are available under SRA BioProject identifier PRJNA1054084 and Biosample identifiers SAMN38882333, SAMN38882334, SAMN38882335, SAMN38882336, SAMN38882337, SAMN38882338, SAMN38882339, SAMN38882340, SAMN38882341, SAMN38882342, SAMN38882343, SAMN38882344 and SAMN38882345. All other data supporting the findings of this study are available from the corresponding author on reasonable request. Source data are provided with this paper.

## Code availability

The macro used to segment DNMT1 foci is available at GitHub (https://github.com/Marteijnlab/DPC-transcription-stress.git)^89^.
