# Supplementary material for: Transcription-coupled DNA–protein crosslink repair by CSB and CRL4CSA-mediated degradation
Source: Nat Cell Biol. 2024 Apr 10;26(5):770–83. doi: 10.1038/s41556-024-01394-y (PMC11098752; doi:10.1038/s41556-024-01394-y)
Supplement: Supplementary file 1 — Reporting Summary [file 41556_2024_1394_MOESM1_ESM.pdf]

nature portfolio

Corresponding author(s): Marteijn

Last updated by author(s): Feb 15, 2024

Reporting Summary

Nature Portfolio wishes to improve the reproducibility of the work that we publish. This form provides structure for consistency and transparency in reporting. For further information on Nature Portfolio policies, see our Editorial Policies and the Editorial Policy Checklist.

Statistics

For all statistical analyses, confirm that the following items are present in the figure legend, table legend, main text, or Methods section.

n/a	Confirmed	
☐	☒	The exact sample size (*n*) for each experimental group/condition, given as a discrete number and unit of measurement
☐	☒	A statement on whether measurements were taken from distinct samples or whether the same sample was measured repeatedly
☐	☒	The statistical test(s) used AND whether they are one- or two-sided<br>*Only common tests should be described solely by name; describe more complex techniques in the Methods section.*
☒	☐	A description of all covariates tested
☒	☐	A description of any assumptions or corrections, such as tests of normality and adjustment for multiple comparisons
☐	☒	A full description of the statistical parameters including central tendency (e.g. means) or other basic estimates (e.g. regression coefficient) AND variation (e.g. standard deviation) or associated estimates of uncertainty (e.g. confidence intervals)
☐	☒	For null hypothesis testing, the test statistic (e.g. *F*, *t*, *r*) with confidence intervals, effect sizes, degrees of freedom and *P* value noted<br>*Give P values as exact values whenever suitable.*
☒	☐	For Bayesian analysis, information on the choice of priors and Markov chain Monte Carlo settings
☒	☐	For hierarchical and complex designs, identification of the appropriate level for tests and full reporting of outcomes
☒	☐	Estimates of effect sizes (e.g. Cohen's *d*, Pearson's *r*), indicating how they were calculated

Our web collection on statistics for biologists contains articles on many of the points above.

Software and code

Policy information about availability of computer code

Data collection	Microscopy data was obtained using commercially available Leica LAS AF software or Carl Zeiss LSM software, as indicated. 4. Flow cytometry: BD LSRFortessa equipped with FACSDiva Software (BD). Colony counter: Automated colony counter from Oxford Optronix Ltd.
Data analysis	Data was analyzed by Leica LAS AF (version 2.7.4.10100) and LAS X (version 3.5.6.21594) software, Carl Zeiss LSM (version 14.0.0.0), ImageJ/Fiji software (version 1.52p) and further processed in Excel (2016) and Prism (version 9.4.0). Maxquant version 1.6.3.3 was used to analyze quantitative proteomics data. Image Studio Lite (Version 5.2.5) was used for Western blot acquisition and analysis Data was plotted and analyzed using GraphPad Prism 9.4.0. The macro used to segment DNMT1 foci is available at GitHub (https://github.com/Marteijnlab/DPC-transcription-stress.git). IGV viewer was used to visualize DPC-seq and RNA-seq data. Flow cytometry data was analyzed using the FlowJo™ software (v.10.8.1) from BD Biosciences.

For manuscripts utilizing custom algorithms or software that are central to the research but not yet described in published literature, software must be made available to editors and reviewers. We strongly encourage code deposition in a community repository (e.g. GitHub). See the Nature Portfolio guidelines for submitting code & software for further information.

Data

Policy information about availability of data

All manuscripts must include a data availability statement. This statement should provide the following information, where applicable:

- Accession codes, unique identifiers, or web links for publicly available datasets
- A description of any restrictions on data availability
- For clinical datasets or third party data, please ensure that the statement adheres to our policy

SILAC-based Pol II quantitative interaction data have been deposited to the ProteomeXchange Consortium via the PRIDE partner repository with the dataset identifier PXD041679. Any other data are available from the corresponding author upon reasonable request. Nascent RNA sequencing data is available under SRA BioProject ID PRJNA1017406, Biosample ID SAMN37395210 and SAMN37395212. DPC-seq sequening data is available under SRA BioProject ID PRJNA1054084, Biosample ID SAMN38882333, SAMN38882334, SAMN38882335, SAMN38882336, SAMN38882337, SAMN38882338, SAMN38882339, SAMN38882340, SAMN38882341, SAMN38882342, SAMN38882343, SAMN38882344, SAMN38882345.
Source data have been provided in Source Data. All other data supporting the findings of this study are available from the corresponding author on reasonable request.

Research involving human participants, their data, or biological material

Policy information about studies with human participants or human data. See also policy information about sex, gender (identity/presentation), and sexual orientation and race, ethnicity and racism.

Reporting on sex and gender	not applicable
Reporting on race, ethnicity, or other socially relevant groupings	not applicable
Population characteristics	not applicable
Recruitment	not applicable
Ethics oversight	not applicable

Note that full information on the approval of the study protocol must also be provided in the manuscript.

Field-specific reporting

Please select the one below that is the best fit for your research. If you are not sure, read the appropriate sections before making your selection.

☒ Life sciences ☐ Behavioural & social sciences ☐ Ecological, evolutionary & environmental sciences

For a reference copy of the document with all sections, see nature.com/documents/nr-reporting-summary-flat.pdf

Life sciences study design

All studies must disclose on these points even when the disclosure is negative.

Sample size	No sample size calculation was performed, sample sizes are similar as to what is common in the field, e.g. PMID: 34108662, PMID: 34108663 and are based on the different experimental procedures e.g. technical difficulty, variation of experiments
Data exclusions	No samples were excluded
Replication	All replications were succesful, all experiments have been excuted at least three times, unless stated differently in the legends. Immunoblots were repeated at least two times.
Randomization	Randomization not relevant as this study does not involve test subjects.
Blinding	Data analyses were performed by software or algorithms and therefore in an unbiased manner, making blinding therefore not applicable.

Reporting for specific materials, systems and methods

We require information from authors about some types of materials, experimental systems and methods used in many studies. Here, indicate whether each material, system or method listed is relevant to your study. If you are not sure if a list item applies to your research, read the appropriate section before selecting a response.

Materials & experimental systems

n/a	Involved in the study
☐	☒ Antibodies
☐	☒ Eukaryotic cell lines
☒	☐ Palaeontology and archaeology
☐	☒ Animals and other organisms
☒	☐ Clinical data
☒	☐ Dual use research of concern
☒	☐ Plants

Methods

n/a	Involved in the study
☒	☐ ChIP-seq
☐	☒ Flow cytometry
☒	☐ MRI-based neuroimaging

Antibodies

Antibodies used

rabbit anti-BRG1 (Abcam ab110641 1:2000), rabbit anti-CSA (Abcam, ab240096, 1:1000), rabbit anti-CSB (Antibodies Online, ABIN2855858, 1:1000), rabbit anti-RPB1 phospho-Ser 2 (Abcam, ab5095, 1:1000) or rat anti-RPB1-phospho-Ser 2 (Chromotek, 3E10, 1:1000), mouse anti-SSRP1 (Biolegend, 609701, 1:10.000), rabbit anti-SPRTN (Invitrogen, PA5-46262, 1:500), mouse anti-SUMO2/3 (Proteintech, 67154-1-1g, 1:1500), mouse anti-Tubulin (Sigma-Aldrich, T5168, 1:5000), rabbit anti-XPA (Genetex, GTX103168, 1:1000), rabbit anti-XPB (Abcam, ab190698, 1:1000), rabbit anti-XPC (Bethyl, A301-112A, 1:2000), rat anti-RPB1-phospho-Ser 5 (Chromotek, 3E8, 1:1000), rabbit anti-USP7 (Bethyl, A300-033A, 1:1000), rabbit anti-VCP (Bethyl, A300-589A, 1:1000), rabbit anti-DDB1 (Novus Biologicals, NBP2-75465, 1:1000), mouse anti-PCNA (Abcam, ab29, 1:200) and rabbit anti-DNMT1 (CST, 5032, 1:200). Secondary antibodies were goat anti-rabbit conjugated to IRdye (Sigma, SAB4600215 (770) or SAB4600200 (680), both 1:10.000), goat anti-mouse conjugated to IRdye (Sigma, SAB4600214 (770) or SAB4600199 (680), both 1:10.000) and goat anti-rat conjugated to IRdye770 (Sigma, SAB4600479, 1:10.000) and Alexa488 or Alexa633 (Invitrogen, 1:1000) .

Validation

Antibodies were validated as indicated on their manufacturer's website, where validated in previous publications of our lab by siRNA/KO experiments or where checked by western blot or immunofluorescence in this manuscript, mostly with a siRNA/KO as control for specificity. All the antibodies used in the manuscript showed bands of expected size.
CSA/ERCC8, abcam ab240096 verified with KO's in supplemental figures 3A and 4D.
CSB/ERCC6, Antibodies-online, ABIN2855858, Santa Cruz, sc376981, verified in our department PMID: 29531219 and verified with KO cells in supplemental figures 3A, 4D and 4L
P-Ser2-RPB1, Chromotek, 3E10, D8L4Y, verified in our lab PMID: 29632207
P-Ser2-RPB1, abcam, ab5095, verified in our lab PMID: 29632207
SSRP1, Biolegend, 609701, verified in our lab PMID: 23973375
Tubulin, Sigma Aldrich, B512, commonly used a loading control in the lab, verified by specific and intense band at correct height
SPRTN, Invitrogen, PA5-46262 PMID: 33567341 and verified in the manuscript Supplemental Figure 4A
XPB, Abcam, ab190698 verified in PMID: 33854616
XPC, Bethyl, A301-121A verified in our department PMID: 32985517
BRG1, Abcam ab110641, verified in our lab PMID: 35750669
rabbit anti-VCP (Bethyl, A300-589A, 1:1000), verified in fig. 8B. and https://www.thermofisher.com/antibody/product/VCP-Antibody-Polyclonal/A300-589A
SUMO2/3, Proteintech, 67154-1-1g, verified with sumo inhibitor in Supplemental Figure 6E
XPA, Genetex, GTX103168, PMID: 35750669 verified in our lab PMID: 53750669 and with a KO in supplemental figure 3A
rabbit anti-USP7 (Bethyl, A300-033A, 1:1000) Verified by siRNA knock-down in Supplemental Figure S7F
rabbit anti-DDB1 (Novus Biologicals, NBP2-75465, 1:1000) Verified by siRNA knock-down in Supplemental Figure S10M
rat anti-RPB1-phospho-Ser 5 (Chromotek, 3E8, 1:1000) verified in our lab PMID: 35750669
mouse anti-PCNA (Abcam, ab29, 1:200) was verified in our lab PMID: 35271816
rabbit anti-DNMT1 (CST, 5032, 1:200) verified in the lab with over expression of DNMT1 .

Eukaryotic cell lines

Policy information about cell lines and Sex and Gender in Research

Cell line source(s)

HCT116 were acquired from Horizon Discovery. HCT116 CSB-mScarlet KI were developed in our lab PMID: 34108662.
MRC-5 sv40 immortalized human lung fibroblast were generated in the lab, the GFP-RPB1 KI were developed our lab PMID: 29632207.
hTert-RPE1 were acquired from ATCC.
HeLa cells (WT, RPB1-K1268R and CSB KO) were generously provided by Tomoo Ogi lab PMID: 32142649.
U2OS WT and USP7 KO cells were generously shared by the Verrijzer lab PMID: 36332031.
WTC-11 human iPSC line (GM25256) containing eGFP-POLR2A were aquired from the Allen Cell Collection, Coriell Institute.
CS-A (CS3BE sv40), CS-B (CS1AN sv40) and UVSS-A (TA-24 sv40) were previously described PMID: 22466611.

Authentication

WT cells were not authenticated. Generated KI or KO cells were authenticated by genotyping, western blot and/or functional assays.

Mycoplasma contamination

All cell lines were routinely tested for mycoplasma and were all negative.

Commonly misidentified lines
(See ICLAC register)

No commonly misidentified cell lines were used in the study.

Animals and other research organisms

Policy information about studies involving animals; ARRIVE guidelines recommended for reporting animal research, and Sex and Gender in Research

Laboratory animals	C. elegans strains used were wild type (Bristol N2), xpa-1(ok698), csa-1(tm5232), csb-1(emc79) and uvs-1(emc80), C. Elegans larvae and adult animals were used as indicated. More specifically, for UV survials L1 stage were irradiated, for FA survivals young adults were allowed to lay eggs for 24 hr on plates.
Wild animals	This study did not involve wild animals.
Reporting on sex	Used C.elegans were hermaphrodites
Field-collected samples	This study did not involve samples collected from the fields.
Ethics oversight	No ethics oversight is required for studies using C.elegans.

Note that full information on the approval of the study protocol must also be provided in the manuscript.

Flow Cytometry

Plots

Confirm that:

☒ The axis labels state the marker and fluorochrome used (e.g. CD4-FITC).

☒ The axis scales are clearly visible. Include numbers along axes only for bottom left plot of group (a 'group' is an analysis of identical markers).

☐ All plots are contour plots with outliers or pseudocolor plots.

☐ A numerical value for number of cells or percentage (with statistics) is provided.

Methodology

Sample preparation	Cells were then harvested by trypsinization, centrifuged for 3 minutes 1200 rpm and resuspended in 500 µl PBS containing 1% FA.
Instrument	Cells were analyzed on a LSRFortessa™ X-20 Cell Analyzer (BD) equipped with FACSDiva Software (BD).
Software	Flow cytometry data was analyzed using the FlowJo™ software (v.10.8.1) from BD Biosciences.
Cell population abundance	Flow cytometry was in this study used to determine Pol II and CSB protein levels by fluorescence quantification. No specific cell types were selected as either isogenic cell lines (hTERT-RPE1 or HCT116 cells) were used. Only gating was used to exclude dead cells and select single cells.
Gating strategy	Gating was used to exclude dead cells based on SCC-A/FSC-A plots and single cells were gated based on FSC-H/FSC-W and subsequently SSC-H/SSC-W

☒ Tick this box to confirm that a figure exemplifying the gating strategy is provided in the Supplementary Information.
