# Supplementary figures and images for: Transcription-coupled DNA–protein crosslink repair by CSB and CRL4CSA-mediated degradation
Source: Nat Cell Biol. 2024 Apr 10;26(5):770–83. doi: 10.1038/s41556-024-01394-y (PMC11098752; doi:10.1038/s41556-024-01394-y)

Figure 3C

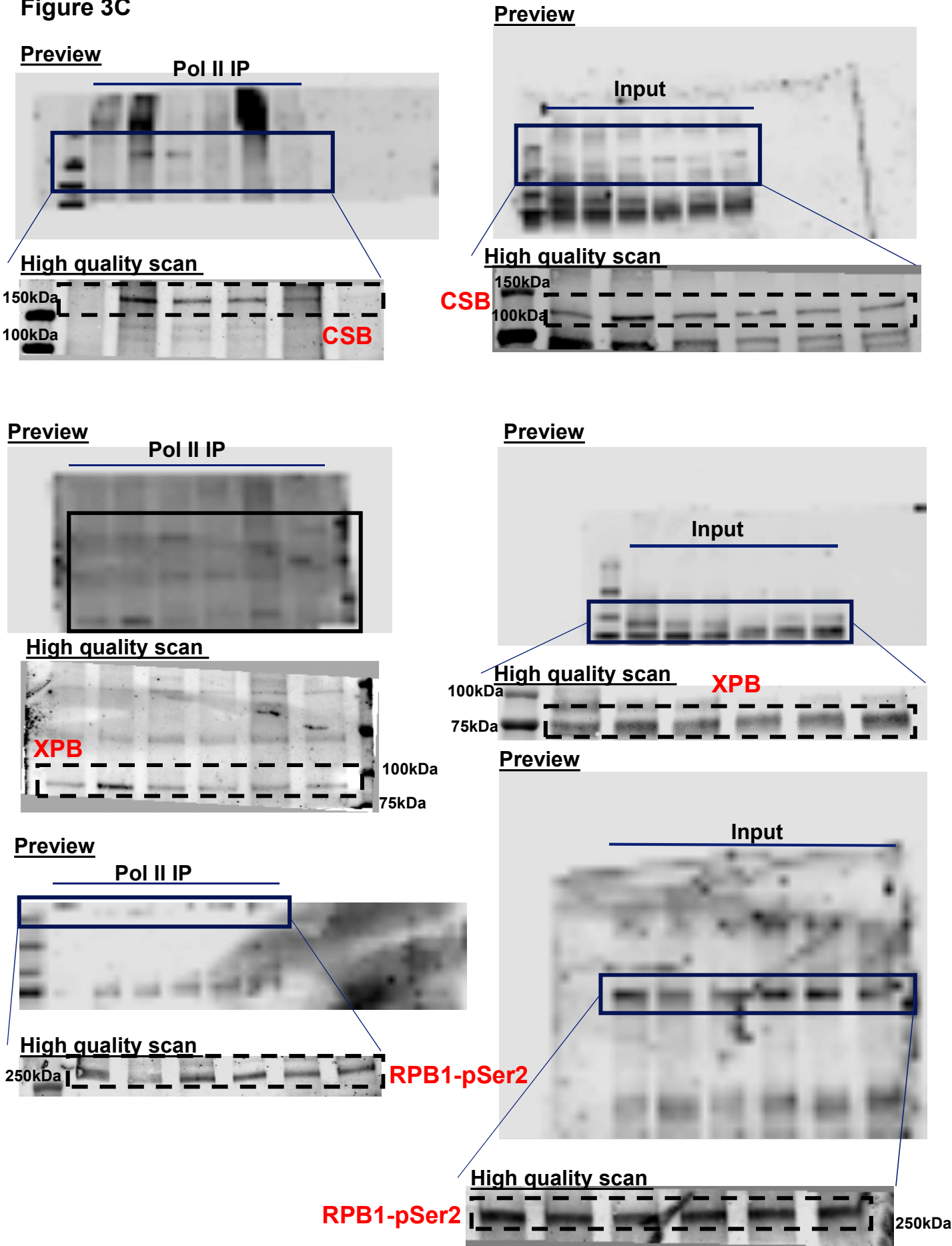

Supplement: Supplementary file 5 — Unprocessed western blots. [file 41556_2024_1394_MOESM5_ESM.pdf]

Figure 7B

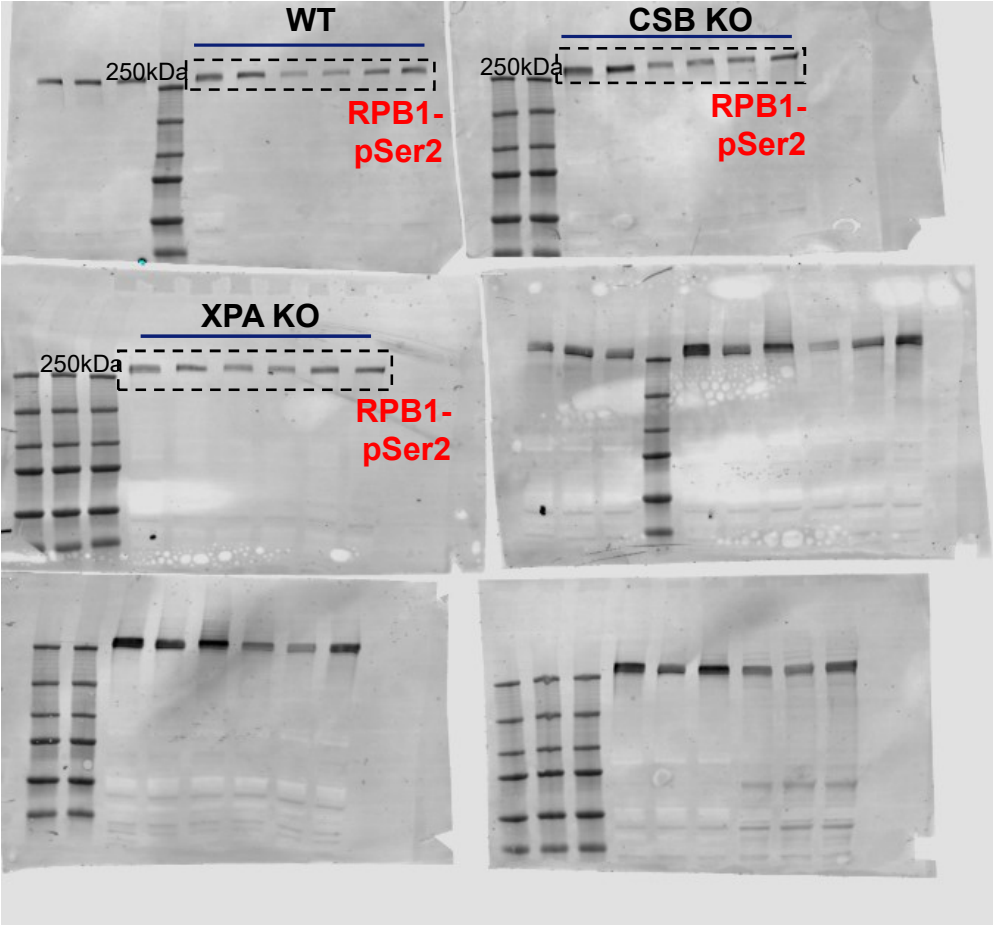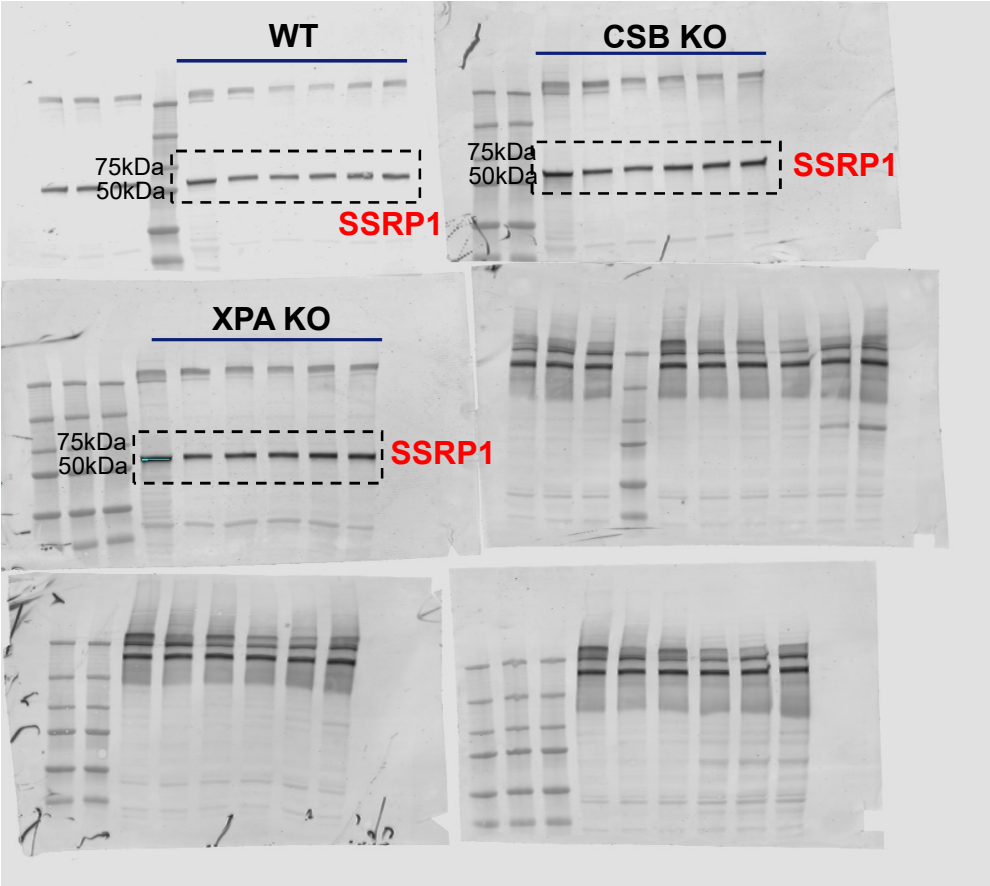

Figure 7B- continued

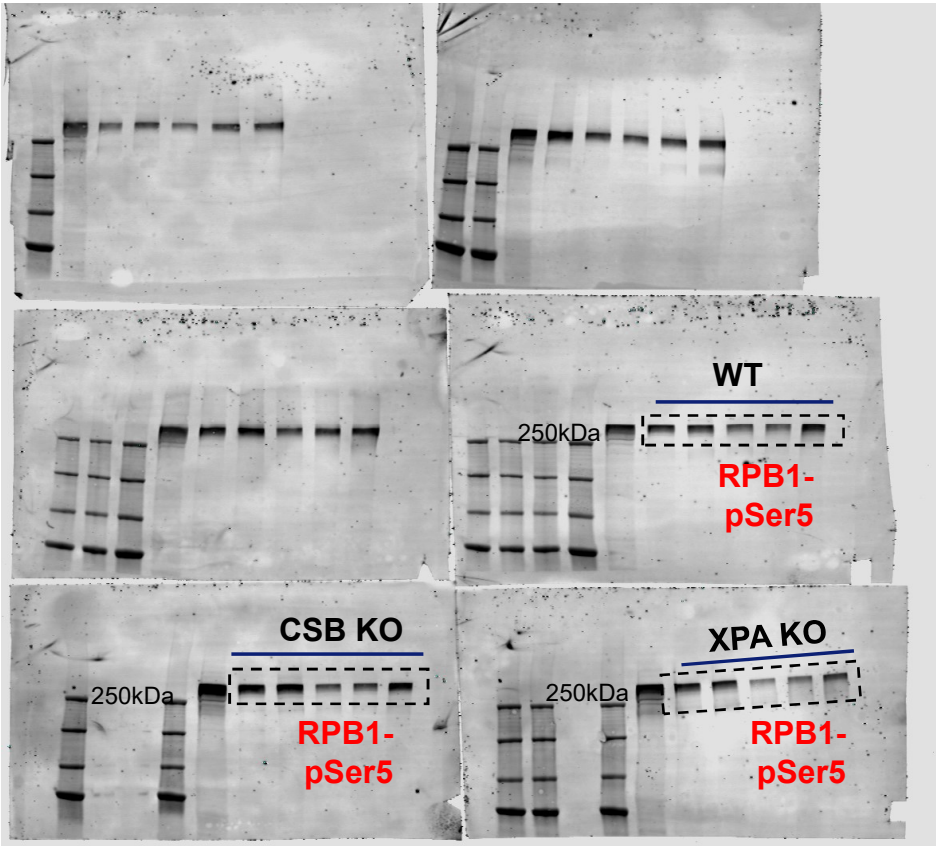

Supplement: Supplementary file 10 — Unprocessed western blots. [file 41556_2024_1394_MOESM10_ESM.pdf]

**Figure 8B**

Preview

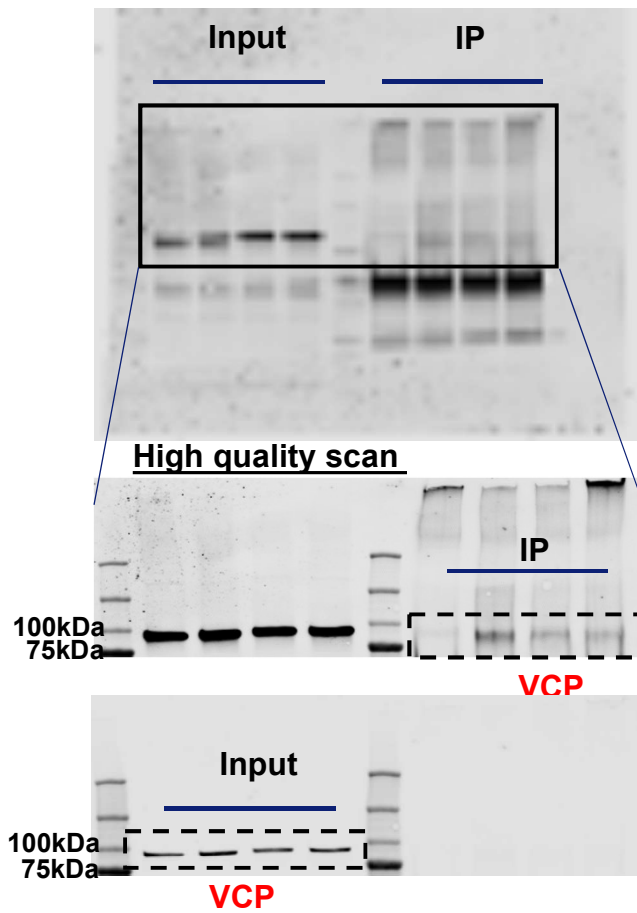

Preview

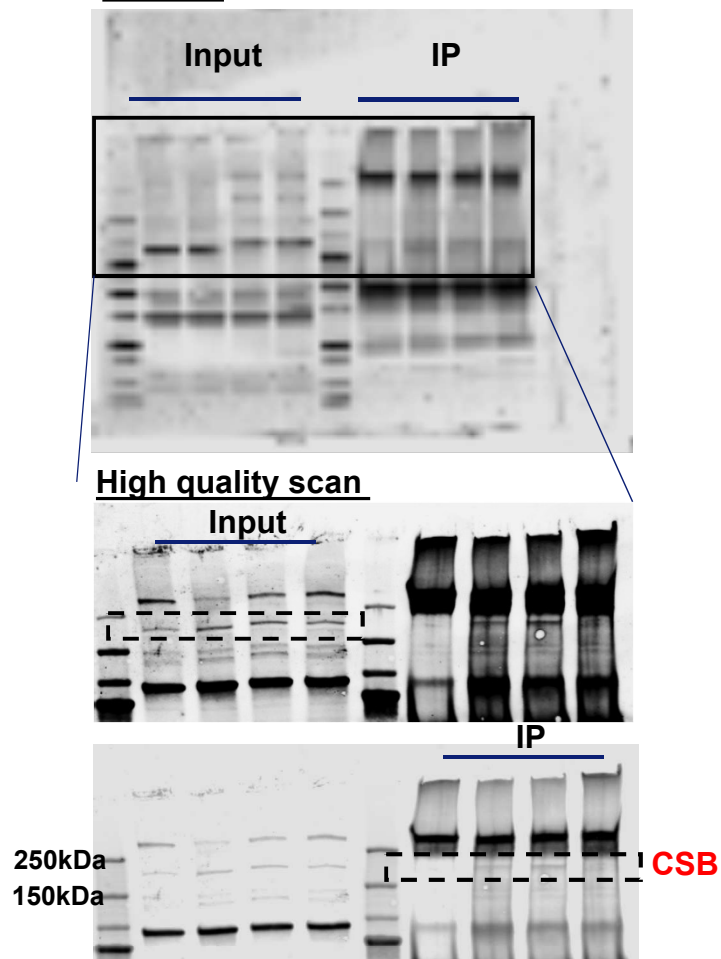

Preview

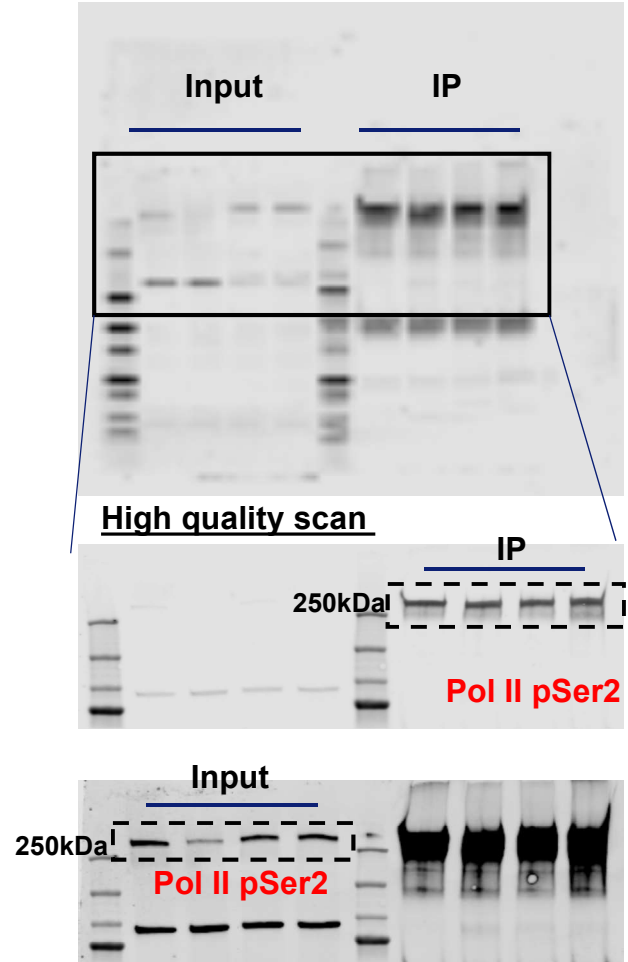

Supplement: Supplementary file 12 — Unprocessed western blots. [file 41556_2024_1394_MOESM12_ESM.pdf]

## Extended Data Figure 1G

### Preview

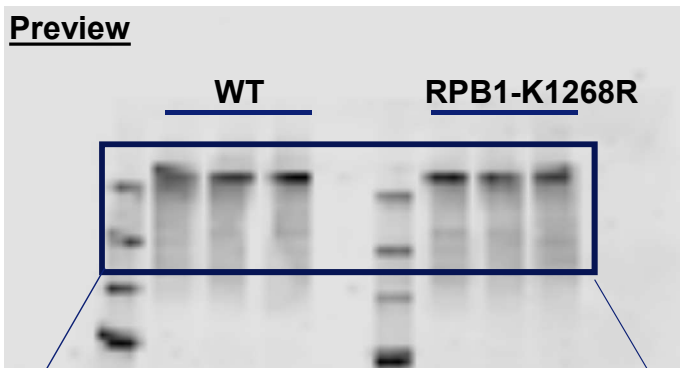

### High quality scan

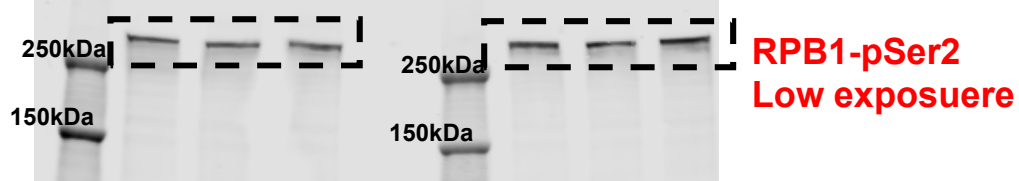

### Higher exposure

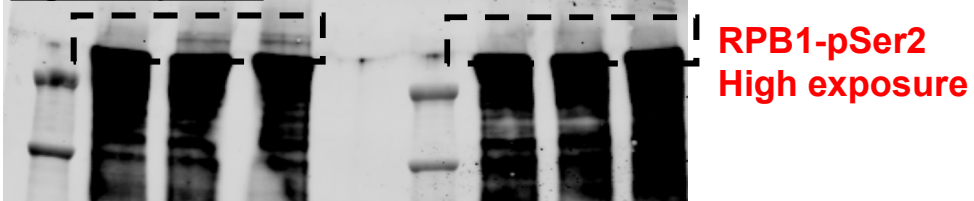

### Preview

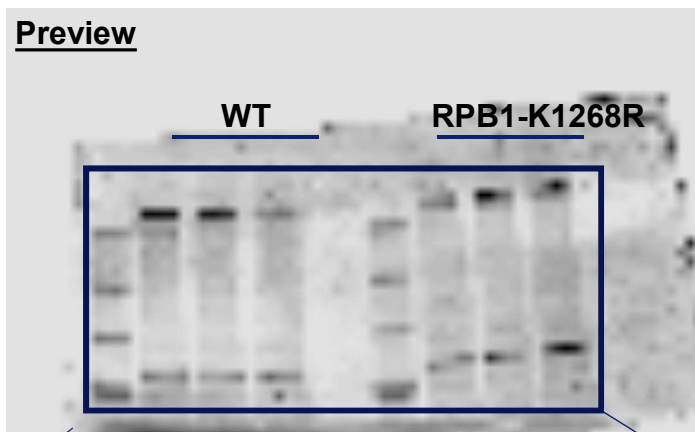

### High quality scan

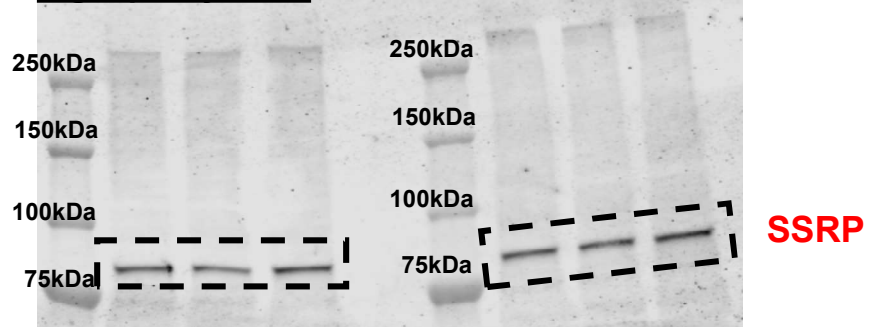

Supplement: Supplementary file 14 — Unprocessed western blots. [file 41556_2024_1394_MOESM14_ESM.pdf]

Extended Data Figure 7A

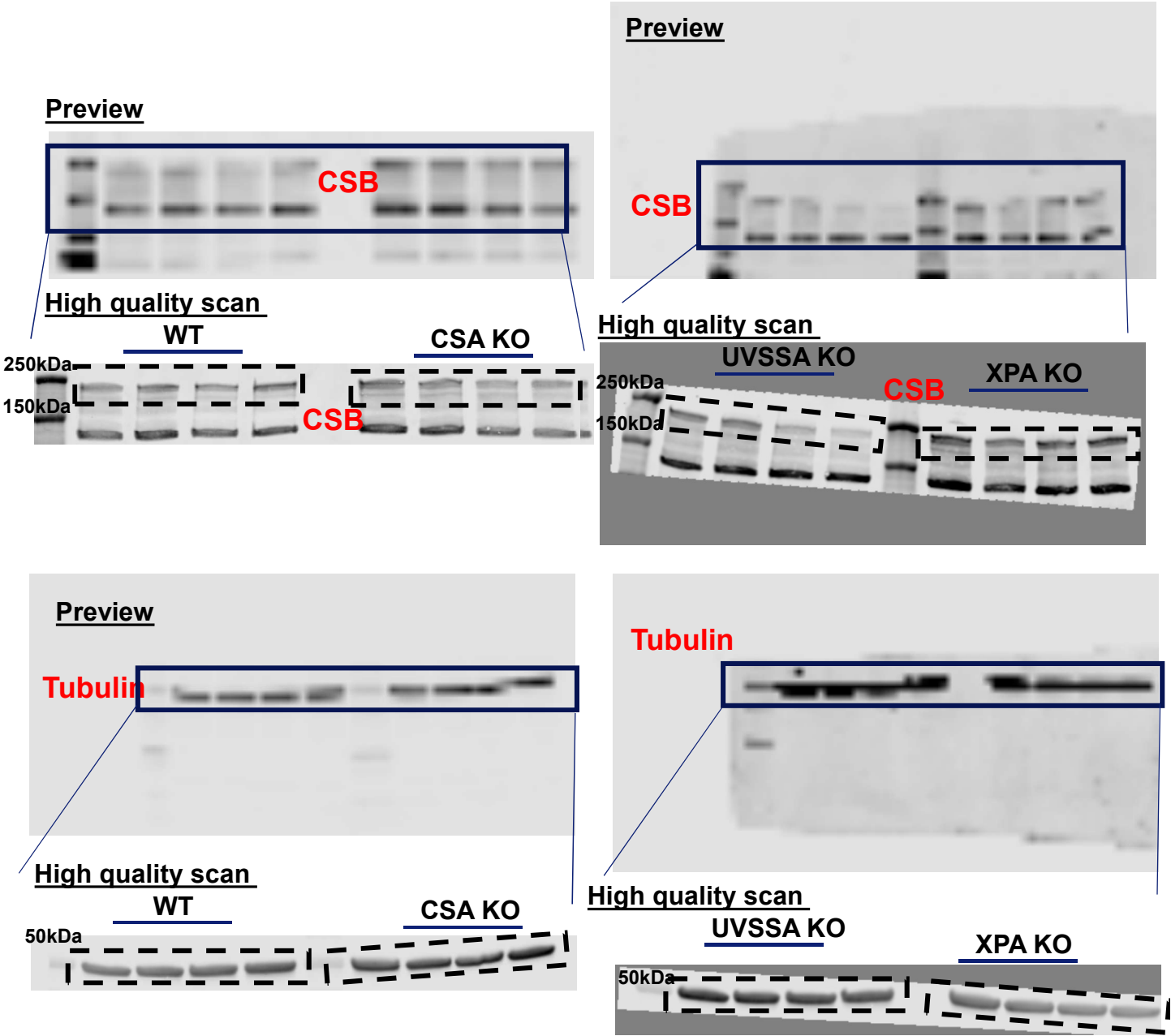

Extended Data Figure 7F

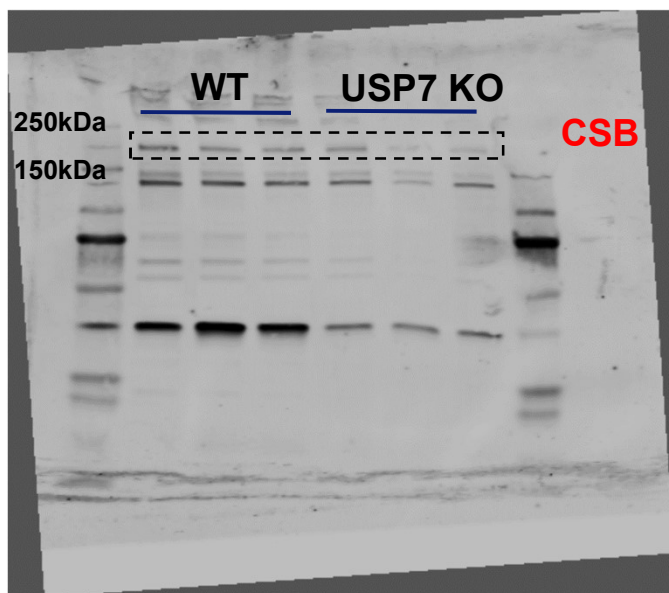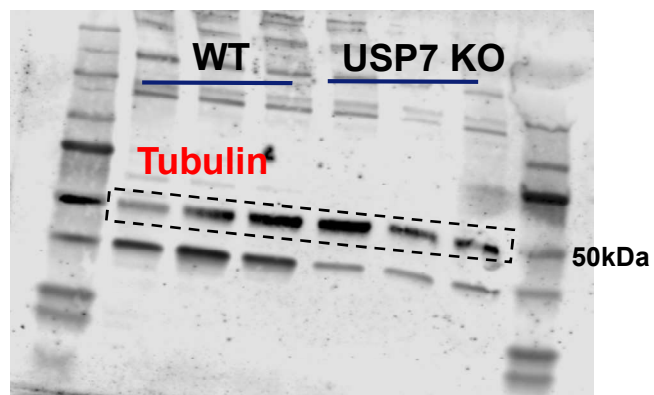

## Extended Data Figure 7G

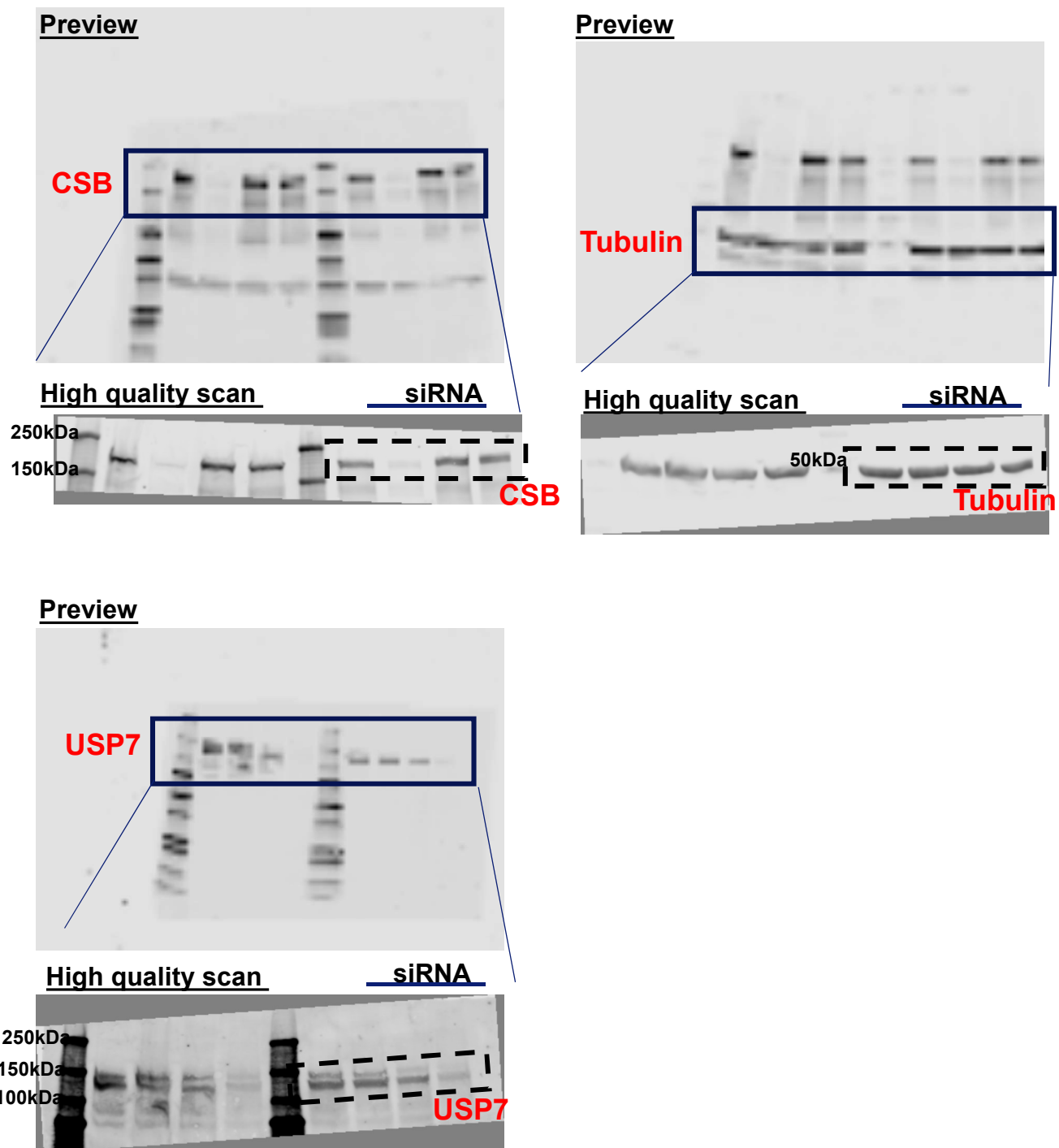

Supplement: Supplementary file 21 — Unprocessed western blots. [file 41556_2024_1394_MOESM21_ESM.pdf]

Extended Data Figure 10I

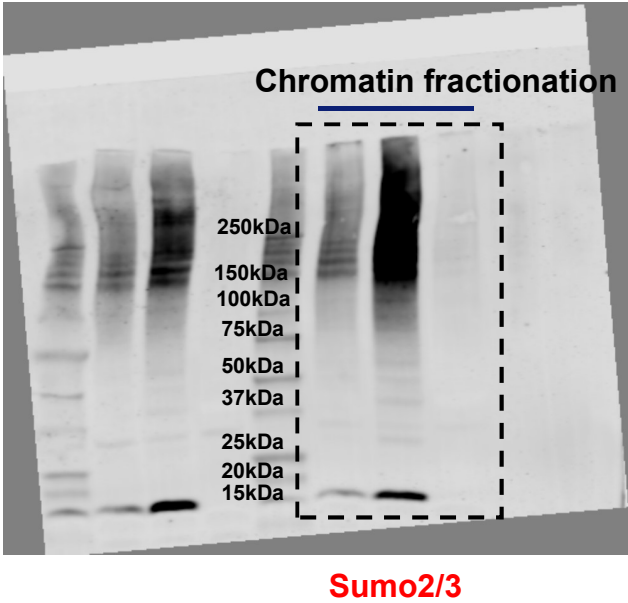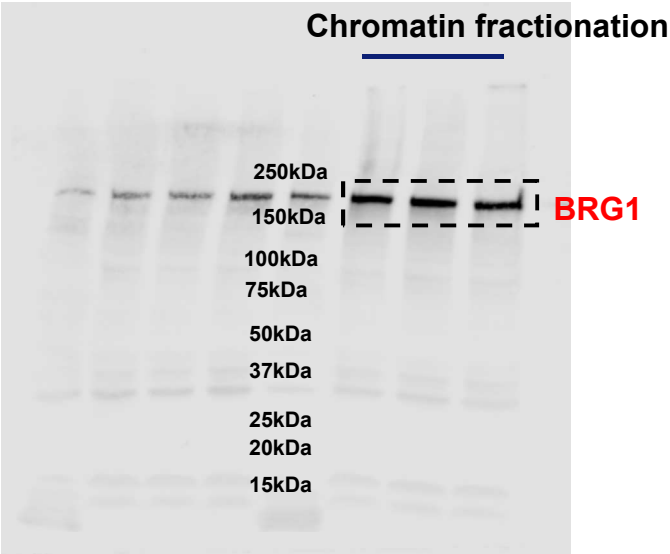

Extended Data Figure S10M

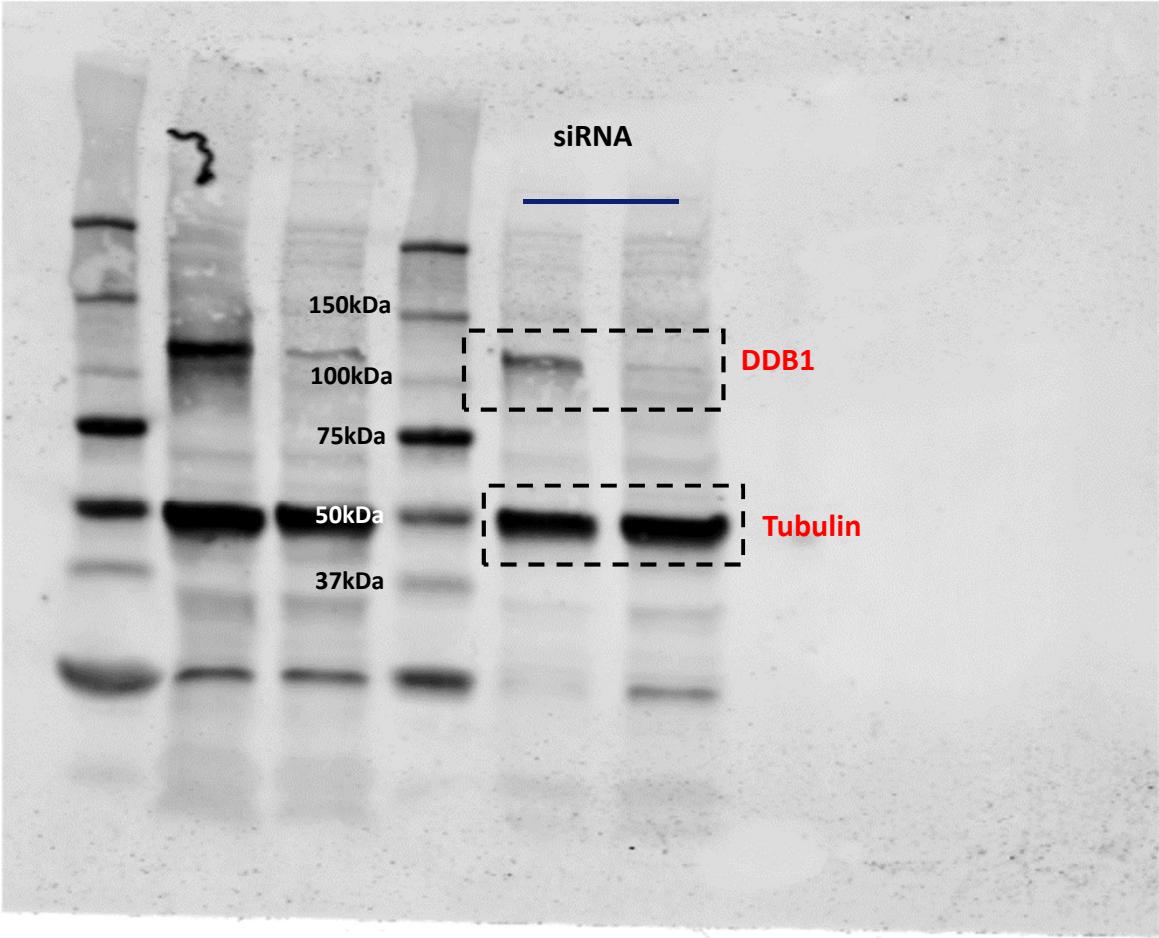

Supplement: Supplementary file 26 — Unprocessed western blots. [file 41556_2024_1394_MOESM26_ESM.pdf]
