# Supplementary material for: Transcription-coupled DNA–protein crosslink repair by CSB and CRL4CSA-mediated degradation
Source: Nat Cell Biol. 2024 Apr 10;26(5):770–83. doi: 10.1038/s41556-024-01394-y (PMC11098752; doi:10.1038/s41556-024-01394-y)

Extended Data Figure 5C

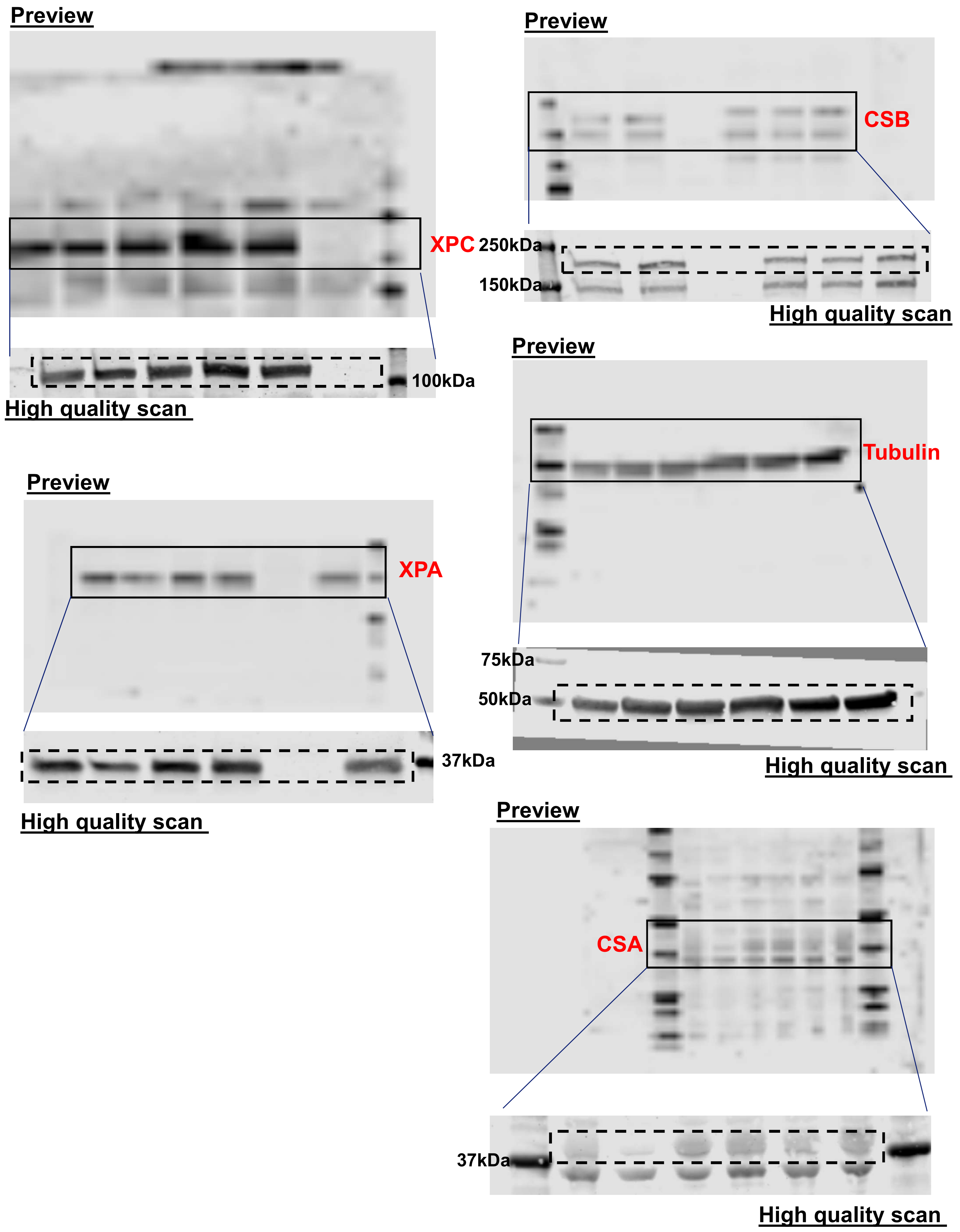

Supplement: Supplementary file 18 — Unprocessed western blots. [file 41556_2024_1394_MOESM18_ESM.pdf]
